# Characterization of the human GnRH neuron developmental transcriptome using a *GNRH1*-TdTomato reporter line in human pluripotent stem cells

**DOI:** 10.1242/dmm.040105

**Published:** 2020-03-13

**Authors:** Carina Lund, Venkatram Yellapragada, Sanna Vuoristo, Diego Balboa, Sara Trova, Cecile Allet, Nazli Eskici, Kristiina Pulli, Paolo Giacobini, Timo Tuuri, Taneli Raivio

**Affiliations:** 1 Stem Cells and Metabolism Research Program, Faculty of Medicine, 00014 University of Helsinki, Helsinki, Finland; 2 Medicum, Faculty of Medicine, 00014 University of Helsinki, Helsinki, Finland; 3 Department of Obstetrics and Gynecology, 00029 Helsinki University Hospital, Helsinki, Finland; 4 Centre for Genomic Regulation, The Barcelona Institute of Science and Technology, Dr. Aiguader 88, 08003 Barcelona, Spain; 5 Inserm, Laboratory of Development and Plasticity of the Neuroendocrine Brain, Jean-Pierre Aubert Research Center, U1172 Lille, France; 6 University of Lille, FHU 1000 Days for Health, School of Medicine, 59000 Lille, France; 7 New Children's Hospital, Pediatric Research Center, 00029 Helsinki University Hospital, Helsinki, Finland

**Keywords:** GnRH neuron, hPSCs, Reporter, Transcriptome, ISL1, Human

## Abstract

Gonadotropin-releasing hormone (GnRH) neurons provide a fundamental signal for the onset of puberty and subsequent reproductive functions by secretion of gonadotropin-releasing hormone. Their disrupted development or function leads to congenital hypogonadotropic hypogonadism (CHH). To model the development of human GnRH neurons, we generated a stable *GNRH1*-TdTomato reporter cell line in human pluripotent stem cells (hPSCs) using CRISPR-Cas9 genome editing. RNA-sequencing of the reporter clone, differentiated into GnRH neurons by dual SMAD inhibition and FGF8 treatment, revealed 6461 differentially expressed genes between progenitors and GnRH neurons. Expression of the transcription factor *ISL1*, one of the top 50 most upregulated genes in the TdTomato-expressing GnRH neurons, was confirmed in 10.5 gestational week-old human fetal GnRH neurons. Among the differentially expressed genes, we detected 15 genes that are implicated in CHH and several genes that are implicated in human puberty timing. Finally, FGF8 treatment in the neuronal progenitor pool led to upregulation of 37 genes expressed both in progenitors and in TdTomato-expressing GnRH neurons, which suggests upstream regulation of these genes by FGF8 signaling during GnRH neuron differentiation. These results illustrate how hPSC-derived human GnRH neuron transcriptomic analysis can be utilized to dissect signaling pathways and gene regulatory networks involved in human GnRH neuron development.

This article has an associated First Person interview with the first author of the paper.

## Abstract (precis)

**Summary:** Here, we generated a *GNRH1*-reporter cell line in hPSCs and investigated transcriptomes of *GNRH1*-expressing neurons and their progenitors, potentially leading to validation of new genes related to GnRH neuron function.

## INTRODUCTION

Hypothalamic gonadotropin-releasing hormone (GnRH) neurons are a central part of the hypothalamus-pituitary-gonadal (HPG) axis that regulates reproduction. GnRH secretion is periodically activated during fetal development, postnatally during ‘minipuberty’, and during adolescence (Kuiri-Hanninen et al., 2014; Young et al., 2019). At puberty, GnRH neurons are gradually reactivated, which increases pituitary gonadotropin and ultimately gonadal sex steroid production. Disruption of GnRH signaling, secretion or GnRH neuron development can lead to congenital hypogonadotropic hypogonadism (CHH), which is characterized by partial or absent puberty, incomplete development of sexual characteristics and often infertility. Patients with CHH combined with anosmia/hyposmia (absent or reduced sense of smell) acquire Kallmann Syndrome (KS) diagnosis (Boehm et al., 2015; Young et al., 2019). Approximately half of the patients can be diagnosed at the molecular level, which suggests the existence of numerous unknown genetic and biological mechanisms controlling puberty.

The first major characterization of human embryonic GnRH neuron localization and migratory route from the frontonasal mesenchyme outside the olfactory and presumptive vomeronasal epithelia to the brain was published in 2016 (Casoni et al., 2016). The newly formed GnRH neurons can only be detected after their delamination from the neuroepithelium into the nasal mesenchyme, and the subpopulation of neuronal progenitors that give rise to GnRH neurons and their gene expression profile are yet to be described (Tucker et al., 2010; Wray, 2010; Forni and Wray, 2015). We have recently reported a protocol for the generation of GnRH-expressing and -secreting neurons from human pluripotent stem cells (hPSCs), in which treatment with FGF8 at the neuronal progenitor stage leads to high expression of anterior neuronal markers such as *FOXG1* and, after further differentiation, these cells give rise to GnRH-expressing neurons (Lund et al., 2016). FGF8 is the growth factor with most potential to have a role in GnRH neuron ontogeny and differentiation, emphasized by the discovery of *FGF8* and *FGFR1* mutations that cause KS and CHH (Chung et al., 2008; Falardeau et al., 2008). The identity of the neuronal progenitor subtype that gives rise to GnRH neurons has not been well characterized. In this study, we generated a GnRH-TdTomato reporter hPSC line using CRISPR-Cas9 technology. The reporter cell line has allowed us to isolate *GNRH1*-expressing neurons by fluorescence activated cell sorting (FACS) and to investigate their mRNA transcriptomes by RNA-sequencing (RNA-seq).

To shed light on the gene expression of the human putative GnRH neuron progenitor subtype and early postmitotic GnRH neurons, we first investigated the differential expression between the FGF8-treated neuronal progenitor pool and TdTomato-expressing GnRH neurons. Second, we used comparison with non-FGF8-treated neuronal progenitors to investigate the putative effects of FGF8-treatment; in both the progenitor pool and in postmitotic GnRH neurons.

The results presented in this study include a large number of differentially expressed genes not previously described in GnRH neurons, which expands the list of candidate regulators of human GnRH neuron development. We have highlighted some potential genes of particular interest in regards to GnRH neuron development and disease modeling. This provides new insight into early stages of human GnRH neuron development and the possibility of identifying markers expressed specifically in GnRH neurons.

## RESULTS

### GNRH1-TdTomato reporter cell line generated using CRISPR-Cas9

**Fig. 1.**
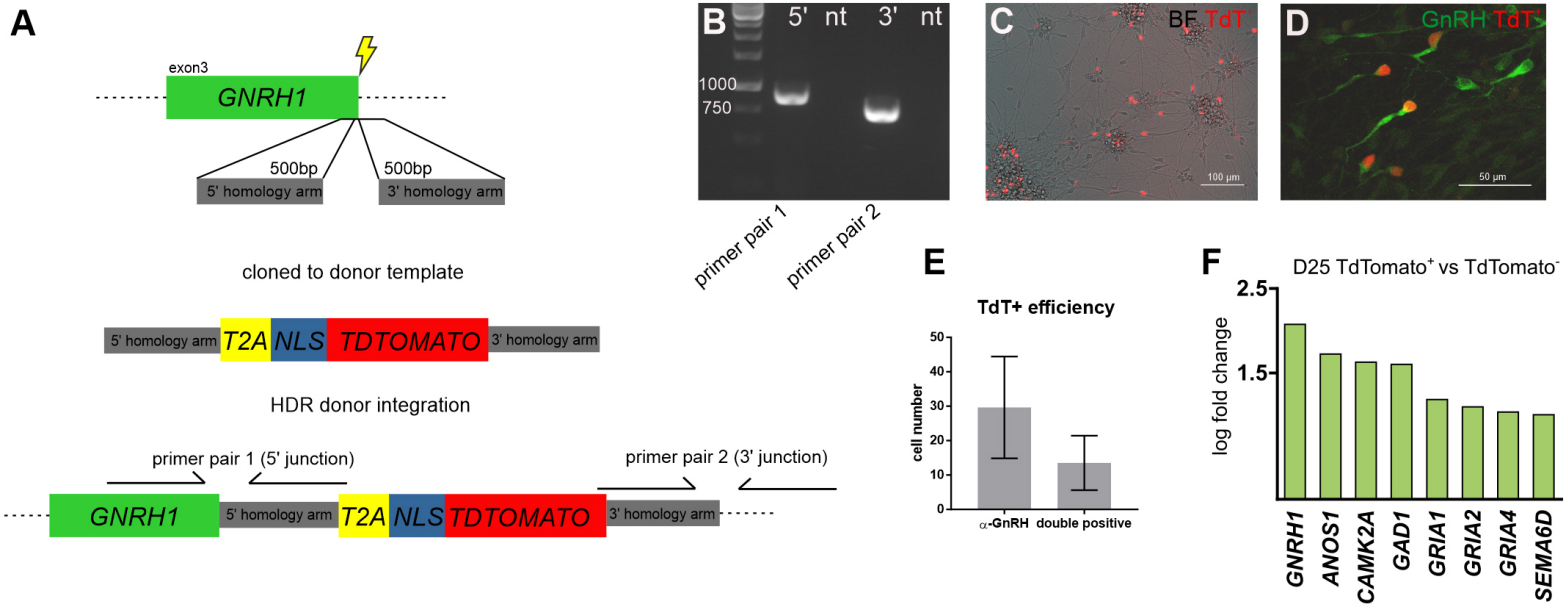
**Generation of GnRH-TdTomato reporter cell line in hPSCs.** (A) Donor template insertion into hPSC genomic DNA was targeted to the last exon of *GNRH1*. The donor template contained T2A self-cleaving peptide and nuclear localization signal (NLS) for cleavage and transportation of TdTomato protein into the nucleus, leaving the GnRH peptide intact. (B) PCR spanning the homologous arm integration into hPSC genomic DNA by the 5′ and 3′ homologous arm junction sites. (C) TdTomato-expressing neurons after differentiation to GnRH neuron lineage. BF, brightfield. (D) Anti-GnRH immunocytochemistry of TdTomato-expressing cells at day 27 of differentiation. (E) Reporter efficiency calculated based on GnRH antibody staining and TdTomato in the same cells (*n*=3, mean±s.d.). (F) RNA-seq of TdTomato-positive versus TdTomato-negative cells (*n*=3) at day (D)25 revealed upregulation of eight genes that have previously been described in GnRH neurons.

To enable the detection and isolation of hPSC-derived GnRH neurons, we generated a *GNRH1*-TdTomato reporter in the previously characterized human induced pluripotent stem cell (iPSC) line HEL11.4 (Mikkola et al., 2013) using CRISPR-Cas9. Before the *GNRH1* stop codon, we knocked-in a reporter sequence encoding a 2A self-cleaving peptide followed by nuclear signal-tagged TdTomato. Homologous recombination of the donor template in the endogenous locus was stimulated using CRISPR-Cas9 targeting adjacently to the *GNRH1* stop codon (Fig. 1A). Altogether, 11 correctly integrated clones were isolated, and one clone (‘A4’) (Fig. 1B) was selected for differentiation into *GNRH1*-expressing neurons according to our previously published protocol (Lund et al., 2016) (Fig. 1C,D). At the end of the protocol, we observed bipolar cells with neuronal morphology that contained the fluorescent TdTomato signal (Fig. 1C), and after immunocytochemistry, we confirmed that the TdTomato signal indeed coincided with anti-GnRH antibody (Fig. 1D). The reporter sensitivity was ∼50% (Fig. 1E), which may reflect differences in GnRH and TdTomato peptide turnover, presence of cells in the culture which are devoid of the TdTomato sequence or allelic switch (Miyanari and Torres-Padilla, 2012). We performed RNA-seq of the TdTomato-positive and negative cells after FACS to investigate their differential expression. We detected 264 differentially expressed genes (*P*-value <0.05) in TdTomato-positive versus -negative samples (*n*=3) and, out of 67 upregulated genes, *GNRH1* had the second highest log fold change (Table S1). In addition to *GNRH1*, the upregulated genes included the KS gene *ANOS1* (Hardelin et al., 1999), as well as *CAMK2A*, *GAD1*, *GRIA1*, *GRIA2*, *GRIA4* and *SEMA6D* (Fig. 1F), expression of which has been previously described in GnRH neurons of animal models (Yang-Feng et al., 1986; Cariboni et al., 2004; Vastagh et al., 2016; Heger et al., 2003; Di Giorgio et al., 2013; Spergel et al., 1999; Bailey et al., 2006; Ebert et al., 2012). These results indicate that TdTomato-expressing neurons successfully mark the formation of GnRH neurons during differentiation from hPSCs. In accordance with these results, the reporter cell line generation was successfully repeated in H9 human embryonic stem cells (ESCs), exhibiting co-expression of TdTomato and anti-GnRH immunopositivity after differentiation on day 25 (Fig. S1).

### RNA-seq reveals differences between FGF8-treated progenitor pool and TdTomato-expressing GnRH neuron mRNA transcriptomes

**Fig. 2.**
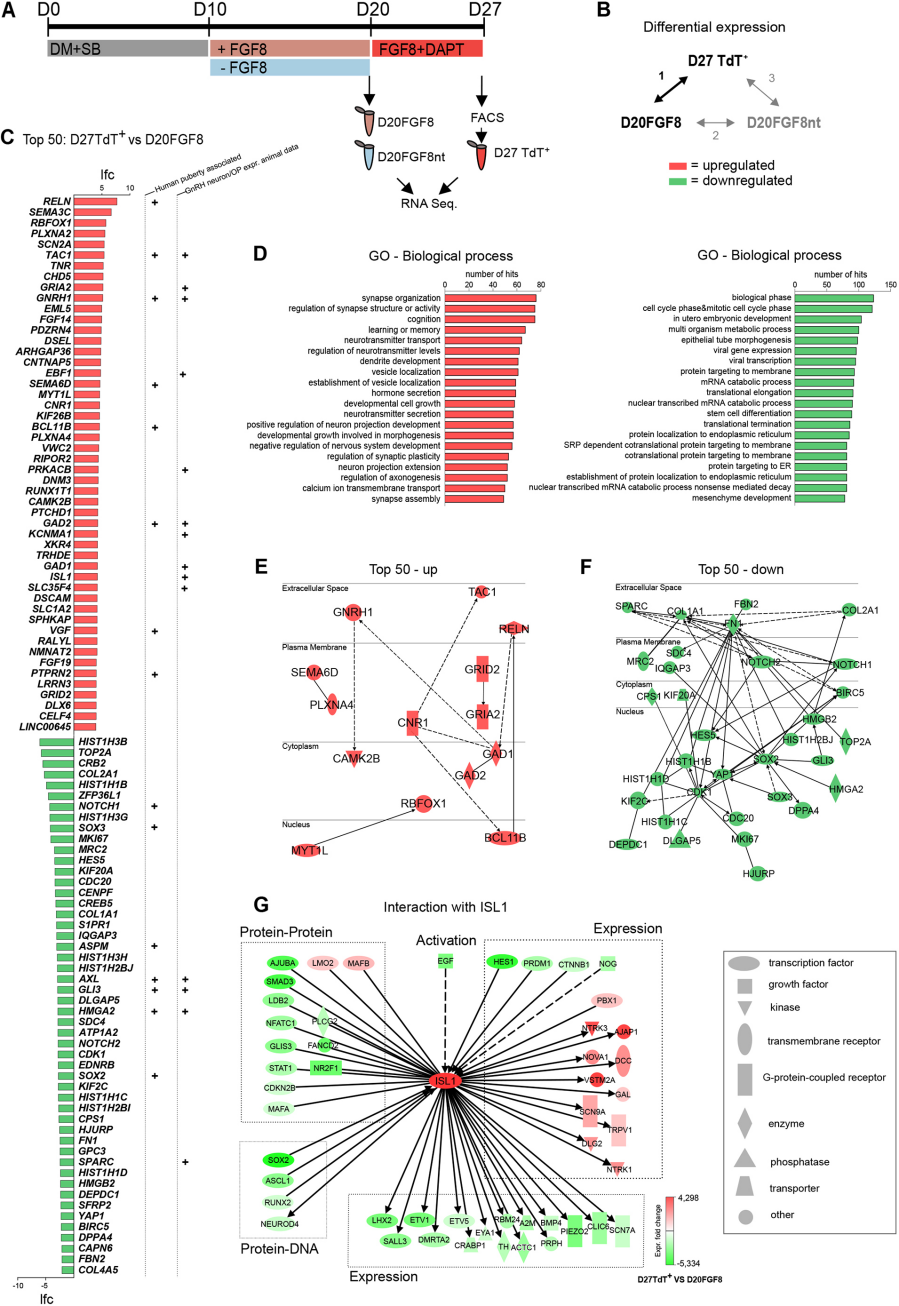
**RNA-seq** **at two time points of differentiation toward GnRH neurons.** (A) The GnRH neuron differentiation protocol begins with 10 days Dual Smad inhibition (DM+SB), followed by 10 days FGF8 treatment to differentiate anteriorly patterned neuronal progenitor cells. To investigate the effect of FGF8 on the RNA profile of the cells, we collected FGF8-treated and non-treated cells at day (D)20. The final seven days of the protocol included Notch inhibition by DAPT. Based on TdTomato reporter expression, we collected the D27 TdTomato-positive samples by FACS sorting on D27. (B) RNA-seq and differential expression analyses were performed between these samples (*n*=4) in three paired comparisons. (C-G) Results from D27TdT^+^ versus D20FGF8 differential gene expression analysis. (C) Transcriptome of GnRH neurons versus FGF8-treated progenitor pool, showing the top 50 upregulated (red, top) and downregulated (green, bottom) genes differentially expressed in D27TdT^+^ versus D20FGF8. Genes previously implicated with an association to puberty timing or expression in, or in close association with, newly formed GnRH neurons (animal models) are indicated (+). (D) Top 20 GO biological processes, and the number of genes per category, enriched in all the significantly upregulated (red, left) and downregulated (green, right) genes D27TdT^+^ versus D20FGF8. IPA was used to draw a pathway containing connections within top 50 upregulated (E) and downregulated (F) genes, and their cytoplasmic localization. (G) IPA pathway of ISL1 upstream and downstream interactions represented in differential expression analysis 1. Lfc, log fold change. See Tables S3,S4 for references associated with panels C, E, F and G.

To further characterize the mRNA transcriptome of TdTomato-expressing GnRH neurons, we analyzed samples from two time points of the differentiation protocol, as indicated in Fig. 2A. First, we included the FGF8-treated neuronal progenitor cell pool (D20FGF8). These contain anteriorly patterned neuronal progenitors, with competence to give rise to GnRH neurons after treatment with Notch inhibitor DAPT (Lund et al., 2016). Second, to investigate the effect of FGF8 treatment, we also included cells not treated with FGF8 (D20FGF8nt) representing neuronal progenitors without specific priming into any distinct neuronal lineage. Finally, we included TdTomato-expressing GnRH neurons (D27TdT^+^) after FACS sorting at day (D)27 (Fig. 2A). We performed differential expression analysis in three sets of comparisons between each sample (*n*=4) (Fig. 2B), to encompass the differences in gene expression between the progenitor pool and differentiated GnRH neurons (analysis 1), as well as the effect of FGF8 treatment during differentiation (analyses 2 and 3).

We first focused on investigating the differences between D20 FGF8-treated progenitors and differentiated GnRH neurons. Comparing D27TdT^+^ with D20FGF8, *GNRH1* was found to be the tenth most upregulated gene (log fold change: 5.2, *P*-value: 1.99×10^−7^) among the 2769 other significantly upregulated and 3692 downregulated genes. Within the top 50 upregulated and downregulated genes (Fig. 2C, Table S2), several have been previously implicated in the timing of puberty, and/or their expression in GnRH neurons has been confirmed in animal models (Table S3). We validated the mRNA expression of 10 out of the top 50 upregulated genes; *RELN*, *SEMA3C*, *RBFOX1*, *PLXNA2*, *SCN2A*, *TAC1*, *GNRH1*, *MYT1L*, *BCL11B*, *ISL1* by qPCR (Fig. S2), and illustrated the presence of RBFOX1, SEMA3C, DSCAM, SCN2A, PLXNA2, substance P (TAC1) and PTPRN2, at protein level in *GNRH1*-expressing neurons after 27 days of differentiation (Figs S3-5).

To compare the differences between all upregulated and downregulated genes, we next performed over-representation analyses to find the most enriched Gene Ontology (GO) biological processes (Fig. 2D). Most over-represented processes in the upregulated genes were related to neuronal maturation, migration or function, including hormone secretion, whereas the downregulated categories were related to stem cell state, cell cycle and morphogenesis. This agrees with our previous observations that the FGF8-treated cell pool at D20 consists of undifferentiated progenitor cells and, by D27, GnRH neurons have acquired neuronal morphology and secrete GnRH decapeptide (Lund et al., 2016; Yellapragada et al., 2019). We next asked whether there are any known interactions within the differentially regulated genes themselves that could represent potential regulatory pathways during GnRH neuron differentiation. We found only 10 previously reported interactions within the top 50 upregulated genes, five of them being direct interactions between SEMA6D-PLXNA4 (protein-protein), GAD1-GAD2 (protein-protein), GRID2-GRIA2 (localization, protein-protein), RELN-BCL11B (expression) and MYT1L-RBFOX1 (protein-DNA, transcription) (Fig. 2E, Table S4). A larger number of interactions were, however, found within the top 50 downregulated genes (Fig. 2F, Table S4), many of which have known roles in sustaining proliferation and repressing differentiation in the progenitor pool, such as NOTCH1, NOTCH2, HES5, SOX2, YAP2 and MKI67 (Herrick et al., 2018; Panaliappan et al., 2018; Huang et al., 2005; Sobecki et al., 2016). We next asked whether some of the upregulated transcription factors have features of potential master regulators, by exhibiting larger numbers of downstream interactions in the data. To explore this effect, we constructed a pathway containing transcription factors in the top 50 upregulated gene list (*EBF1*, *MYTL1*, *BCL11B*, *RUNX1T1*, *ISL1* and *DLX6*) and their reported downstream interaction to the top 500 upregulated genes (Fig. S2). Out of these transcription factors, *ISL1*, a LIM/homeodomain family transcription factor with a known role as a specifying transcription factor in early spinal motor neurons (Rhee et al., 2016; Cave and Sockanathan, 2018), displayed the most known downstream interactions within the top 500 upregulated genes (Fig. S6). We searched for reported interactions with *ISL1* represented in the RNA-seq data, and found ‘protein-protein’, ‘protein-DNA’, ‘expression’ and ‘activation’ interactions upstream and downstream of *ISL1* within the differentially expressed genes (Fig. 2G). Together, the transcriptome data and knowledge-based interaction pathways offer prediction of some of the putative mechanisms involved in GnRH neuron differentiation, and suggest a role for *ISL1* in regulating the expression of effector genes during GnRH neuron differentiation.

### ISL1 in hPSC-derived GnRH neurons and human fetal GnRH neurons

**Fig. 3.**
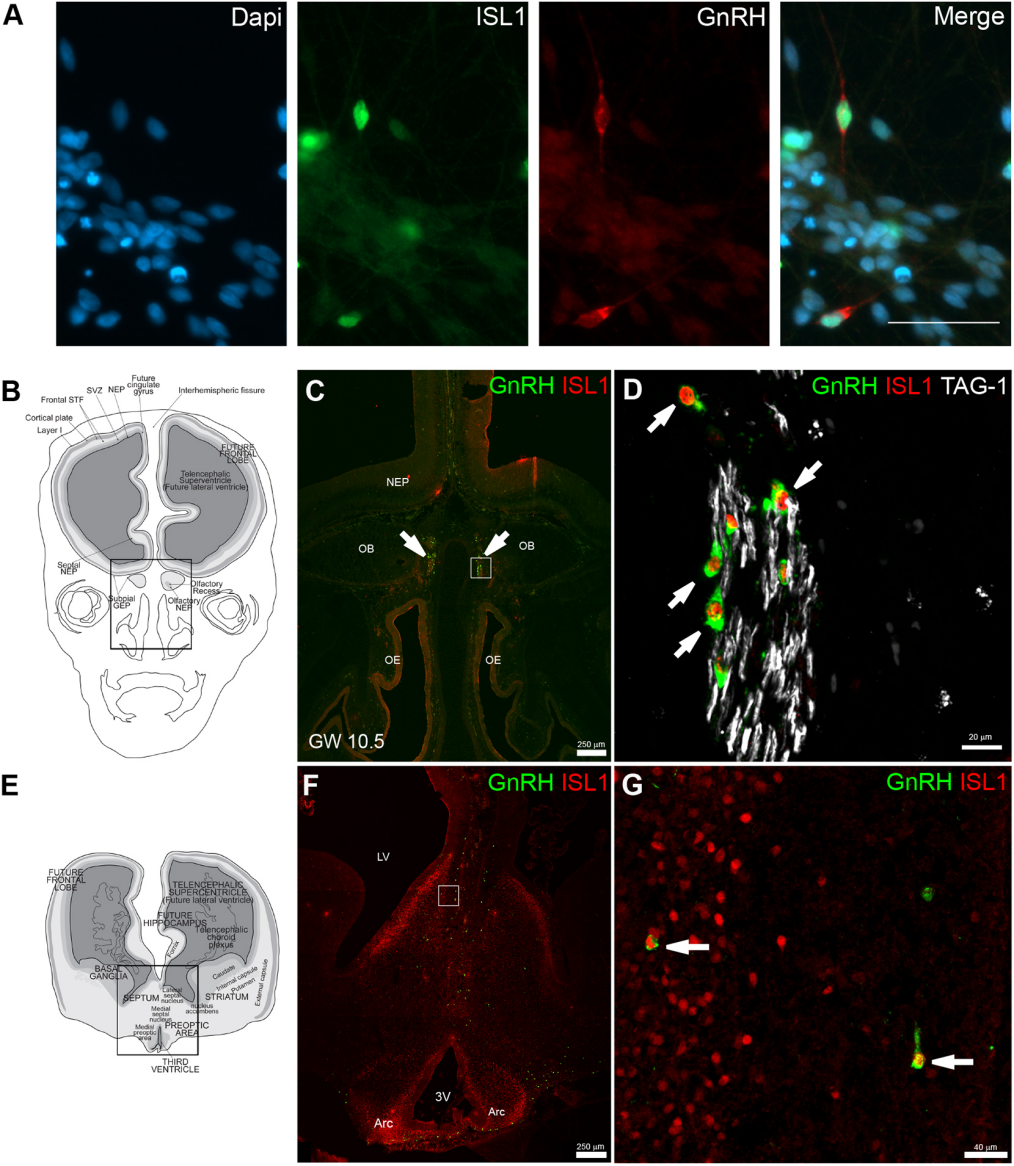
**ISL1 expression in hPSC-derived and human fetal GnRH neurons.** (A) Immunocytochemical staining with ISL1 and GnRH antibodies at day 27 of differentiation showed nuclear staining of ISL1 in GnRH-positive neurons. Result has been replicated in 3 experiments. (B) Schematic of a GW10.5 human fetus head (coronal view). Box indicates the area represented in immunohistochemical staining in C and D. (C,D) GnRH (green), ISL1 (red) and TAG-1 (white) expression in a coronal section of a GW10.5 fetus. ISL1 is expressed in GnRH neurons located in the nose and entering the forebrain (olfactory bulbs, OB). D shows a magnified view of the boxed area in C. (E) Schematic of a GW10.5 human fetus brain (coronal view). Box indicates the area represented in immunohistochemical staining in F and G. (F,G) ISL1 (red) is expressed in GnRH neurons that have migrated into the forebrain (septum). G shows a magnified view of the boxed area in F. White arrows show GnRH/ISL1 double-labeled cells. Arc, arcuate nucleus; OE, olfactory epithelium; LV, lateral ventricle; NEP: neuro-epithelium; 3V: third ventricle. Scale bars: 50 µm in A; 250 µm in C,F; 20 µm in D; 40 µm in G.

To confirm ISL1 protein expression in human GnRH neurons, we performed immunocytochemistry for ISL1. In accordance with the RNA-seq results, nuclear ISL1 was present in α-GnRH-positive neurons at D27 of the differentiation (Fig. 3A). ISL1 was not detected in D20 progenitor cells, which suggests that ISL1 becomes expressed in postmitotic neurons. We then analyzed by triple-immunofluorescence the expression pattern of ISL1 in human fetuses [10.5 gestational weeks (GW), *n*=2] together with the expression of GnRH and transient axonal glycoprotein 1 (TAG-1; also known as contactin 2; Fig. 3B-G), which highlights the axonal scaffold for the migration of GnRH neurons (Yoshida et al., 1995; Casoni et al., 2016).

All GnRH-immunoreactive neurons expressed ISL1 throughout the migratory pathway, both in the nasal compartment (Fig. 3B-D) and in the brain septal area (Fig. 3E-G). These results indicate that ISL1 is expressed in fetal GnRH neurons after their differentiation, and expression of ISL1 persists during the migration to the hypothalamus.

### Presence of genes implicated in CHH in mRNA profiles of hPSC-derived GnRH neurons

**Fig. 4.**
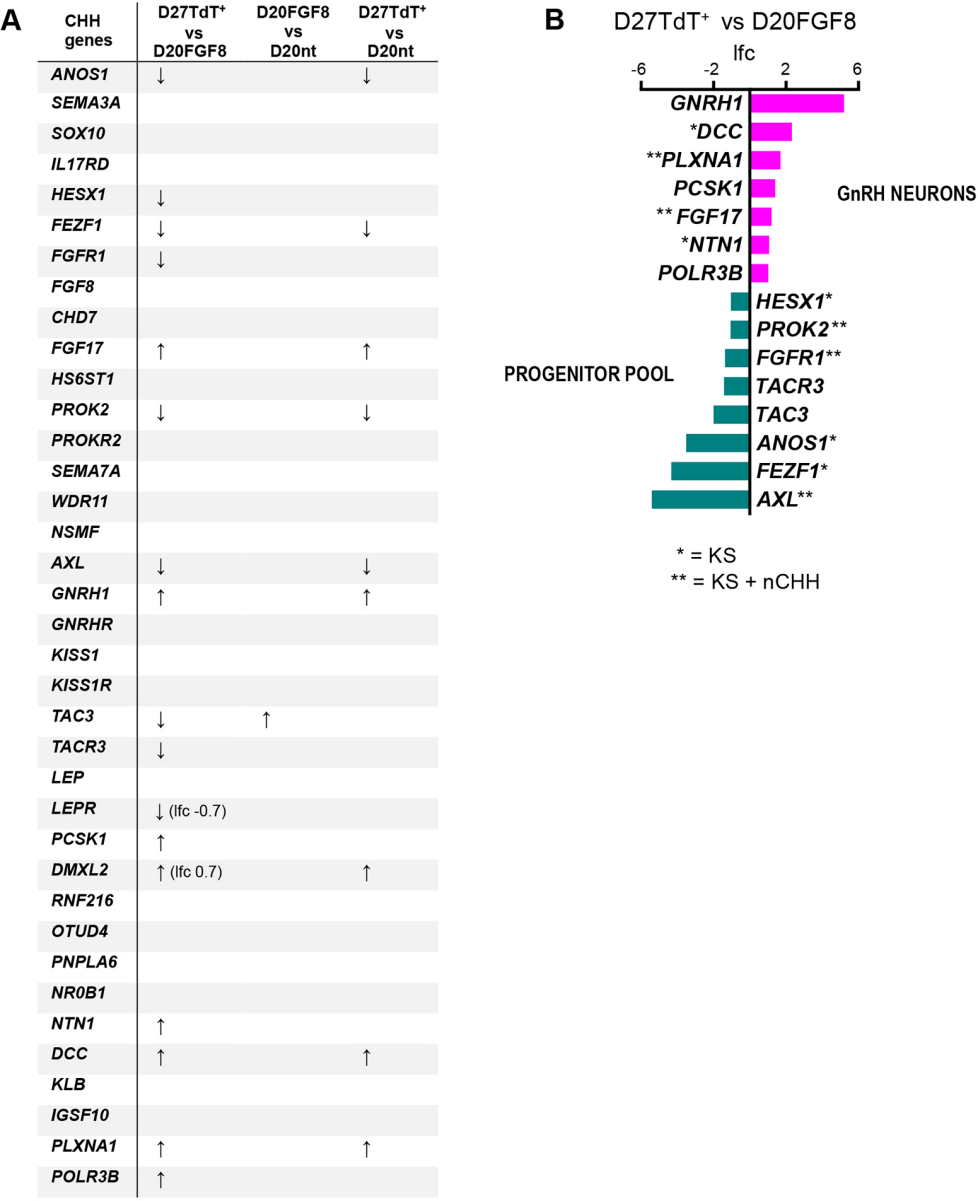
**CHH-associated genes differentially expressed in TdTomato-expressing GnRH neurons and their progenitors.** (A) Table of known CHH-associated genes and their occurrence in the RNA-seq analyses. Arrows up or down indicate upregulated and downregulated genes, respectively. Differential expression was detected for *LEPR* and *DMXL2*, although their log fold change values (lfc) were below the threshold that was set as significant (>1 and <−1), and are therefore marked in the table. No entry indicates that differential gene expression was not detected. (B) Known CHH-associated genes present in D27TdT^+^ versus D20FGF8 differential gene expression. Positive lfc indicates that the gene was significantly upregulated in GnRH neurons, negative lfc indicates that the gene was significantly upregulated in the progenitor pool. Asterisks indicate association of gene mutations to KS (featuring anosmia) or normosmic (n) CHH.

To compare the current knowledge about CHH genetics with this model, we searched our RNA-seq data for a list of 37 reported CHH genes (Fig. 4A), including the genes associated with both KS (i.e. anosmic phenotype) and normosmic CHH (Boehm et al., 2015; Bouilly et al., 2018; Howard et al., 2016; Xu et al., 2017; Richards et al., 2017; Kotan et al., 2018). We found 15 validated CHH genes differentially expressed in D27TdT^+^ versus D20FGF8 (Fig. 4B). Netrin 1 (NTN1) and its receptor deleted in colorectal cancer (DCC) are known to be involved in GnRH neuron migration during embryonic development, and DCC expression has been reported in GnRH neurons (Low et al., 2012; Bouilly et al., 2018; Schwarting et al., 2004). Also plexin-A1 (PLXNA1), receptor of class 3 semaphorins, is involved in GnRH neuron embryonic migration in mice (Cariboni et al., 2007; Marcos et al., 2017). To our knowledge, expression of *PCSK1*, *FGF17* and *POLR3B* has not been previously reported in GnRH neurons specifically. *PCSK1* and *POLR3B* are associated with normosmic CHH (Pepin et al., 2018; Richards et al., 2017), but *FGF17* mutations are associated with KS and anosmia, which implies a role during early GnRH neuron migration from the olfactory placodes as well (Valdes-Socin et al., 2014). Interestingly, eight of these 15 genes were among the downregulated genes, which implies higher expression in the progenitor pool. A possible explanation is a role for these genes in the earlier phases of differentiation, or within the niche of developmentally related neuronal progenitors. In accordance, *HESX1*, *FGFR1*, *ANOS1* and *FEZF1* expression has been reported in the nasal placodes (Carvalho et al., 2003; Chung et al., 2008; Hirata et al., 2006; Kotan et al., 2014), *PROK2* and *AXL* along the GnRH neuron migratory route (Pitteloud et al., 2007; Allen et al., 2002; Pierce et al., 2008), and *TAC3* and *TACR3* are expressed by hypothalamic kisspeptin/neurokinin B/dynorphin (KNDy) neurons (Navarro and Tena-Sempere, 2011). In conclusion, several genes implicated in CHH and associated with early stages of GnRH neuron development were present in this data, including nine genes that are associated with KS and anosmia, suggesting involvement in embryonic GnRH neuronal migration from the olfactory placode.

### Genes expressed after treatment with FGF8 in progenitors and GnRH neurons

**Fig. 5.**
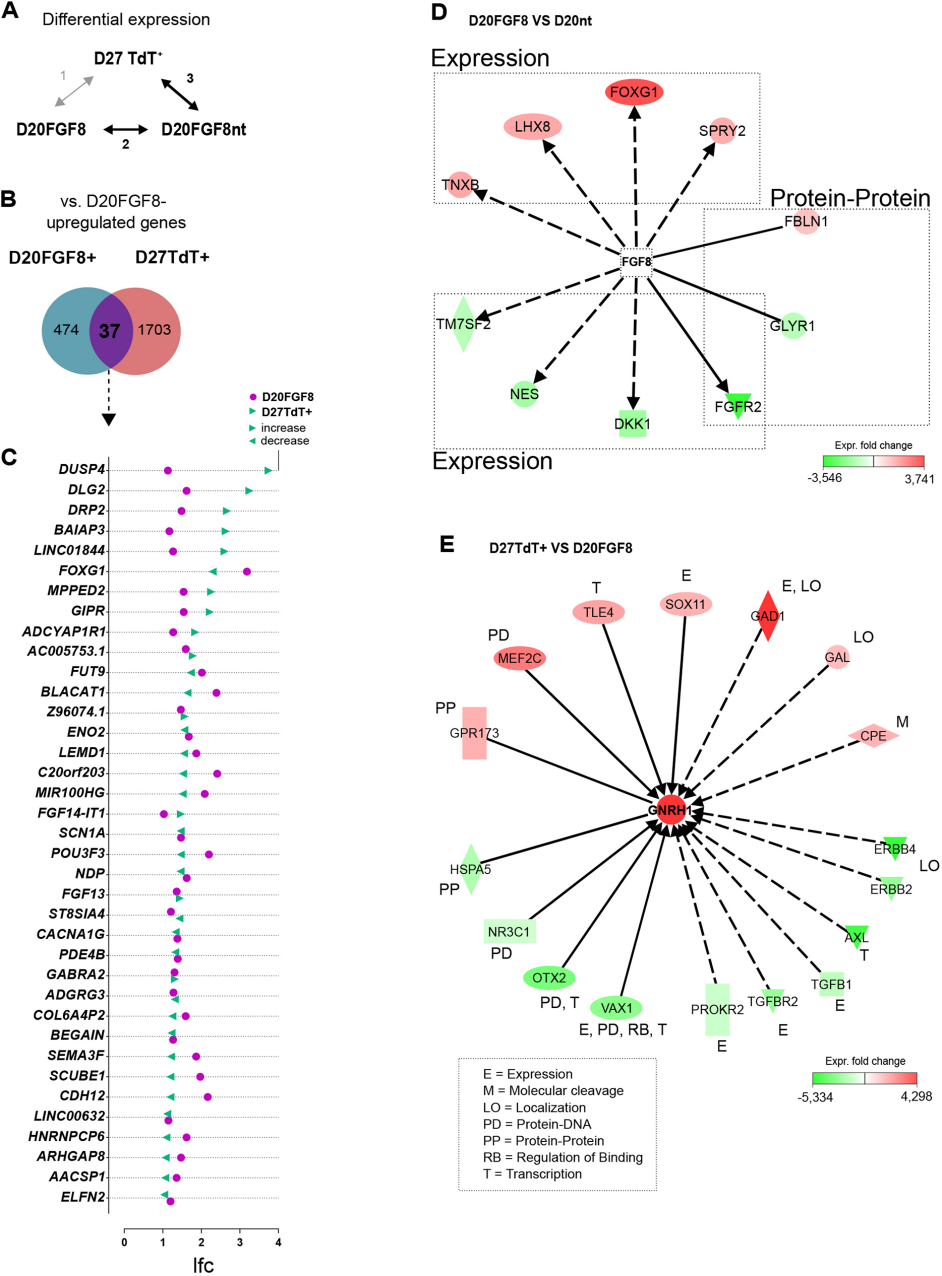
**Overlap in genes upregulated by FGF8-treatment in D20 progenitors and GnRH neurons.** (A) Examination of the differential gene expression in analyses of D20FGF8 versus D20FGF8nt and D27TdT^+^ versus D20FGF8nt, to describe the effect of FGF8 treatment. (B) There was an overlap of 37 genes upregulated in both analyses. (C) List of 37 overlapping genes that were upregulated and their respective log fold changes. (D) IPA pathway analysis of known downstream effectors of FGF8, present in differential expression analysis D20FGF8 versus D20FGF8nt. (E) IPA pathway analysis of known genes upstream of *GNRH1* present in D27TdT^+^ versus D20FGF8nt. Shapes in E are as indicated in Fig. 2. See Table S8 for references associated with interaction data presented in panels D and E.

At D20, FGF8-treated progenitor cells organize in neuronal rosettes that express proliferation marker Ki67 (MKI67) and neuronal stem cell/progenitor cell marker SOX2. Cells grown without FGF8 usually have fewer and smaller neuronal rosettes, and already show presence of some bipolar neuron-like cells, but do not produce GnRH-expressing cells at the end of the protocol (Lund et al., 2016). In accordance, FGF8 has a known role in promoting stem and progenitor cell survival and proliferation (Tucker et al., 2010; Diez del Corral et al., 2002). To identify the effects of FGF8 treatment on mRNA expression, we next examined differential expression between FGF8-treated and non-treated samples (Fig. 5A). We performed differential gene expression analysis of D20FGF8 versus D20FGF8nt, to investigate the effects of FGF8 in the progenitor pool, and differential gene expression of D27TdT^+^ versus D20FGF8nt, to determine putative effects of FGF8-treament which are also sustained in postmitotic GnRH neurons. Finally, to identify overlap within significantly upregulated genes in FGF8-treated samples, we combined the results of upregulated genes from these two analyses (Fig. 5B). We found 37 upregulated and 61 downregulated genes that were overlapping in both D20FGF8 versus D20FGF8nt and D27TdT^+^ versus D20FGF8nt (Table S7). Out of the 37 overlapping genes upregulated in both analyses (Fig. 5C), seven genes were also found to further increase expression in TdTomato-expressing GnRH neurons (*DUSP4*, *GIPR*, *BAIAP3*, *DRP2*, *MPPED2*, *LINC01844* and *DLG2*), suggesting that these genes have been upregulated in the FGF8-treated neuronal progenitors and their expression has remained and/or increased after GnRH neuron specification. The most upregulated gene in D27TdTomato-expressing GnRH neurons in this comparison, *DUSP4*, is a member of the dual specificity protein phosphatase family, which negatively regulate members of MAPK superfamily (Niwa et al., 2007) and also regulate neuronal differentiation in mouse ESCs by linking the Mapk/Erk and calcium signaling pathways (Kim et al., 2015). Also the transcription factor *FOXG1* was among the common upregulated genes, and supports our previous observation of high *FOXG1* mRNA and protein expression in neuronal progenitors following FGF8 treatment (Lund et al., 2016). Furthermore, one of these 37 overlapping genes, *FGF13*, was recently reported to be enriched in mouse GnRH neurons by RNA-seq (Burger et al., 2018), and also described as a CHH candidate gene (Quaynor et al., 2016).

To explain the role of FGF8 in inducing gene expression changes that could ultimately lead to specification of GnRH neurons, we used Ingenuity Pathway Analysis (IPA) to predict the upstream effect of FGF8 based on known interactions. In D20FGF8 versus D20FGF8nt, we found 10 known downstream effectors of FGF8 signaling present in the data, also including the aforementioned *FOXG1* (Fig. 5D, Table S8). Based on these downstream effectors, FGF8 action within the neuronal progenitor cells is likely implemented in part by upregulated expression of *FOXG1*, *LHX8*, *SPRY2* and *TNXB*. *FGFR2* downregulation was represented in both D20FGF8 versus D20FGF8nt and D27TdT^+^ versus D20FGF8nt (Table S7). FGF8 acts through cognate receptors FGFR1 and FGFR3, and cell type-specific regulation of receptor-type expression by FGF8 has been demonstrated (Mott et al., 2010). This regulation of receptor expression is likely to further influence the activation of downstream intracellular pathways.

When compared to the D20FGF8nt samples, TdTomato-expressing GnRH neurons, which are differentiated from the FGF8-treated progenitor pool, revealed upregulation of genes associated with several GO biological processes related to neuronal differentiation and function (Fig. S7), and we next asked whether some of these genes have any reported direct interactions upstream of *GNRH1*. We found *TLE4*, *SOX11*, *MEF2C*, *GAL*, *GAD1*, *CPE* and *GPR173* among the upregulated genes (Fig. 5E, Table S8). SOX11 has been demonstrated to activate *GNRH1* gene expression in murine immortalized GnRH neuronal cells (Kim et al., 2011), whereas MEF2C is involved in suppression of GnRH expression during embryonic migration (Allen et al., 2000, 2002). GPR173 is a G protein-coupled receptor for phoenixin peptide, which was recently found to increase GnRH expression in a murine immortalized hypothalamic GnRH neuron cell line (Treen et al., 2016). In conclusion, some of the candidate genes presented in these results may play important roles in the regulation of *GNRH1* expression during GnRH neuron differentiation and maturation processes.

## DISCUSSION

During human fetal development, GnRH neurons differentiate from the nasal neuroepithelial compartment, which is derived from the olfactory placodes, and begin their migration to the hypothalamus (Casoni et al., 2016). These newly formed postmitotic embryonic GnRH neurons express *GNRH1* and neuronal markers such as DCX, β-tubulin III and DNER, as they emerge just outside the presumptive vomeronasal organ, which contains SOX2 and Ki67-positive putative precursor cells. The questions of the identity and distinctive markers for the neuronal progenitor subtype still remain to be answered. The primary objectives of using hPSCs for differentiation of GnRH neurons is to model GnRH neuron development and provide a platform to study the biological mechanisms that regulate GnRH neuron differentiation. CRISPR-Cas9 genome editing has recently become one of the central methods in biomedical research, with benefits for stem cell research, disease modeling and studying differentiation of specialized cell types including GnRH neurons (Yellapragada et al., 2019). To compare our hPSC-derived model for human GnRH neuron development to previously performed characterization of GnRH neurons, we compared our data to previously reported genes from animal models and found that many of these were indeed present among the differentially expressed genes, which included genes implicated in CHH, GnRH neuron and olfactory placode markers. This is of particular interest as the disease mechanisms of CHH and milder forms of pubertal delay are largely unknown, and disease-modeling with hPSCs is expected to reveal such mechanisms. For example, with the aid of CRISPR-mediated gene editing and activation (CRISPRa) (Weltner et al., 2018) it will be possible to investigate how altered expression of a single gene or a set of genes enriched in our transcriptome data affect human GnRH neuron specification and GnRH secretion (Lund et al., 2016). Conversely, researchers working with exome and whole genome sequencing data from CHH patients might find our data useful when attempting to deduce the pathogenic and causative variants in their data sets. Finally, the use of patient-derived iPSCs in combination with CRISPR genome editing will allow investigation into the relative contribution of multiple rare sequence variants acting either alone or in combination (i.e. oligogenecity; Young et al., 2019) affecting GnRH cell phenotype.

On the other hand, when we searched for previously published interactions within the differentially expressed genes between TdTomato-expressing GnRH neurons and the progenitor pool, we noticed a substantially lower number of interactions within the group of upregulated genes. The small number of reported interactions itself reflects the complexity of mechanisms governing GnRH neuron development that are yet to be described. Among the top 50 upregulated genes in TdTomato-positive GnRH neurons were *SEMA6D* and *PLXNA4* (Fig. 2C,E), with a previously reported protein-protein interaction (Toyofuku et al., 2004). In accordance, semaphorin-plexin signaling is known to be crucial for embryonic GnRH neuron migration (Lettieri et al., 2016). *RBFOX1*, one of the upregulated genes in TdTomato-expressing neurons, also detected by immunocytochemistry (Fig. S3) has not been previously described in GnRH neurons, but is known to regulate neuronal development and subtype specification by alternative splicing (Wamsley et al., 2018), and its interactor MYT1L has been described as a pan neuronal transcription factor that represses non-neuronal fates during differentiation (Mall et al., 2017; Hu et al., 2013). A role for BCL11B in the regulation of the expression of reelin (*RELN*) has been previously suggested, during the differentiation of mouse medium spiny neurons (Arlotta et al., 2008). Interestingly, *BCL11B* has been presented as a candidate gene to regulate puberty timing (Day et al., 2017), and Bcl11b expression has been reported in mouse vomeronasal neurons during embryonic development (Enomoto et al., 2011), whereas RELN has been implicated in migration of GnRH neurons (Dairaghi et al., 2018; Cariboni et al., 2005).

Among the highly upregulated genes in TdTomato-expressing GnRH neurons was the transcription factor *ISL1*, and its expression in human postmitotic GnRH neurons was confirmed in fetal tissues. ISL1 is known to regulate gene expression by forming protein complexes with transcription factors such as LIM domain-containing factors (Caputo et al., 2015; Gay et al., 2000). The protein-protein and protein-DNA interactions of ISL1 have the specific outcome of activating downstream neuron subtype-specific genes (Lee et al., 2016; Rhee et al., 2016; Zhang et al., 2018). This raises the possibility that ISL1, in co-operation with other yet-to-be-defined transcription factor(s), functions as a master regulator in GnRH neurons during their terminal differentiation. We found 49 reported interactions between ISL1 and the genes represented in the RNA-seq data. These included for example PBX1, a suggested repressor of *GNRH1* expression (Rave-Harel et al., 2004). Together, this advocates for ISL1 involvement in regulating gene expression during GnRH neuron differentiation, which is likely to be cell type-specific, and further studies are needed to determine its mechanism of action during GnRH neuron development.

FGF8 signaling through FGFR1 is indispensable for the neurogenesis of GnRH neurons (Kawauchi et al., 2005; Falardeau et al., 2008; Chung et al., 2008), suggesting an important role for FGF8 during GnRH differentiation. The olfactory placode in mice, the neurogenic niche that is known to give rise to GnRH neurons, lies in an area in the ventral olfactory placode that is patterned by mesenchymal and epidermal sources of FGF8, BMP and noggin (Forni et al., 2013; Forni et al., 2011). The neurogenic permissive area is first established by the mesenchymal-derived BMP/TGFβ inhibition, in which FGF8 acts as a pro-neurogenic factor by having an indirect effect on the positioning of the GnRH neuron progenitor niche, by modulation of signals from the mesenchymal patterning centers (Forni et al., 2013). We have found that FGF8 treatment stimulates the hPSC-derived neuronal progenitor's ability to produce GnRH neurons *in vitro*, but the specific downstream influences of FGF8 treatment are not fully resolved. We previously showed that *FOXG1* expression becomes upregulated during the FGF8 treatment (Lund et al., 2016). Accordingly, we have now observed that the genes differentially expressed following FGF8 treatment also included *FOXG1*. *FOXG1* expression is known to be regulated by FGF8 in the developing brain (Shimamura and Rubenstein, 1997; Storm et al., 2006; Diez del Corral et al., 2002; Lund et al., 2016), and *FOXG1* expression in the neurogenic niche in the nasal pit has been reported as one of the requirements for proper GnRH neuron development (Garaffo et al., 2015; Duggan et al., 2008). FGF8 treatment of the differentiating progenitor pool should therefore lead to downstream changes in gene expression, which subsequently lead to the establishment of a GnRH neuron progenitor lineage during hPSC differentiation. In an effort to identify some of the downstream effector genes, we compared the differential expression analyses of FGF8-treated versus non-treated samples and found 37 genes as likely downstream targets responding to FGF8 signaling. *DUSP4* stood out with the largest fold-increase in TdTomato-expressing cells, at D27 when GnRH neurons have differentiated. DUSPs have known roles in negative regulation of the MAPK/ERK pathway (Caunt and Keyse, 2013) and have been reported in the regulation of neuronal development and axonal growth through modulation of ERK activity (Finelli et al., 2013). Dusp4 expression reportedly increases during neuronal differentiation in mouse ESCs, where its knockdown affected the ERK activity. This resulted in changes in calcium signaling pathway activity, which was suggested to be the mechanism that regulates neurite growth and neural marker expression in these cells (Kim et al., 2015). In our transcriptome data, calcium ion transmembrane transport was one of the top 20 enriched biological processes in TdTomato expressing GnRH neurons (Fig. 2D), which suggests calcium signaling being actively regulated in these cells. A potentially interesting aspect is to further investigate whether calcium signaling serves as a mechanism through which FGF8 and Notch pathways regulate neuronal differentiation. Besides, another dual specificity protein phosphatase, DUSP6, could play an important role during GnRH neuron development. Mutations in *DUSP6* have been identified in normosmic CHH and KS patients, when candidate genes were screened in patient samples based on similar expression patterns and protein interaction within the FGF8 pathway ‘synexpression group’ (Miraoui et al., 2013). Indeed, Dusp6 expression has been reported in the nasal placodes during mouse embryo development (Dickinson et al., 2002). In addition to negative regulation of MAPK signaling (Li et al., 2007), DUSP6 has been suggested to modulate calcium-dependent neurotransmitter release homeostasis by downregulation of L-type calcium channel Cav1.2 expression (Mortensen, 2013). Considering these findings, the DUSP-family proteins may be players in modulating FGF8 activity during GnRH neuron differentiation.

In conclusion, we present data describing the hPSC-derived GnRH neuron transcriptome, and as a contribution towards better understanding of GnRH neuron development and differentiation, we expect that these data will lead to broader validation of new genes pertinent to GnRH neuron function.

## MATERIALS AND METHODS

### Generation of GNRH1-TdTomato reporter knock-in to HEL11.4

The GNRH1-TdTomato reporter cell line was generated using CRISPR-Cas9 genome editing (Balboa et al., 2017) with the strategy to target the stop codon at the *GNRH1* gene for insertion of the donor template GNRH1_T2A_NLS_TdT_PGK_puro. The donor template was designed with 577 (5′) and 599 bp (3′) homologous arms to introduce the donor sequence into the genome by homologous recombination. The homologous arms were cloned by PCR from the genomic DNA of the human embryonic stem cell line H9 with primers containing BamH1 and Nhe1 (5′ homologous arm) and Ascl1 and Xba1 (3′ homologous arm) restriction sites for cloning. The fragments were PCR purified (NucleoSpin Gel and PCR Clean-up, Macherey-Nagel) and digested. The fragments were then purified from agarose gel and ligated to the donor template vector T2A-2xNLS-TdTomato-PGK-Puro. Guide RNA sequences were designed using crispr.mit.edu/Guides and the guide cassettes were amplified and purified as described previously (Balboa et al., 2017). Guide sequence and homologous arm primers sequence are provided in Supplementary Materials and Methods.

### Electroporations and clonal expansion of knock-in iPSC clones

HEL11.4, a previously characterized human iPSC line [Biomedicum Stem Cell Centre (BSCC)] (Mikkola et al., 2013) were grown in Stem Pro (Thermo Fisher Scientific) and were dissociated using Accutase (Life Technologies) and resuspended in cold 5% fetal bovine serum (FBS)/PBS. Three electroporations were performed using Neon Transfection system (Life Technologies) according to the manufacturer's instructions, with a total of 6×10E6 cells with 18 µg CAG-Cas9 (gift from Diego Balboa, Biomedicum Stem Cell Centre, Finland; Addgene plasmid #89995), 6 µg pUC-GNRH1-T2A-NLS-TdT-PGKPuro and 1.5 µg guide RNA using a pre-optimized program (1100V, 20 ms, 2 pulses) and plated onto Matrigel (Corning) matrix-coated dishes with 10 μM ROCK inhibitor (Y-27632 2HCl, Selleckchem) in Stem Pro (Life Technologies). On the following day, medium was changed to Stem Pro with 5 μM ROCK inhibitor. ROCK inhibitor was removed after 48 h, and selection with 0.15 µg/ml Puromycin (Sigma-Aldrich) was started 72 h after electroporation. Medium was refreshed daily until colonies established. Colonies were picked on Matrigel-coated 96-well plates in Stem Pro+5 µM Rock inhibitor ∼7-10 days after electroporation. All cell lines have been tested for mycoplasma contamination.

### Screening of knock-in clones

For collection of DNA samples, cell colonies in 96-well plates were treated with 0.5 mM EDTA (Invitrogen) for 4 min, after which the EDTA was removed and replaced with 100 µl Stem Pro+10 µM ROCK inhibitor, and the cells were detached from the plate by scraping with a 10 µl pipette tip. From each well, 50 µl of cell suspension was left in a flat-bottomed 96-well plate for continued culture and freezing, whereas the other 50 µl was moved to a V-bottom plate. The V-bottom plate was centrifuged at 2300 rpm (889 ***g***) for 3 min, and supernatant removed. Then 60 µl PCR Direct lysis buffer (Viagen Biotech) and 5 µl Proteinase K (Viagen Biotech) was pipetted to each well, and the plate was incubated at 55°C for 2 h and 85°C for1 h, and frozen at −20°C until use. Touchdown PCR for detection of integration of the 5′ and 3′ homology arms was performed using the primers and conditions listed in Supplementary Materials and Methods.

The reporter cell line generation was repeated in the hPSC line H9 (WiCell) according to the methods described above. One successful clone (‘C11’; Fig. S1) was used, together with the original clone, during validation of the RNA-seq results.

### GnRH-neuron differentiation of knock-in clones

Three confirmed knock-in clones were thawed for GnRH neuron differentiation which was performed as described in Lund et al. (2016). Briefly, cells were expanded in Stem Pro on Geltrex-coated (Life Technologies) culture dishes. Differentiation started at 90% confluence, in N2B27+2 µM Dorsomorphin (Selleckchem) and 10 µM SB431542 (Sigma-Aldrich). On day 10, cells were passaged, and medium changed to N2B27+100 ng/ml FGF8 (Peprotech). At day 20, cells were passaged, and medium was changed to N2B27+100 ng/ml FGF8+20 µM DAPT (Selleckchem), until FACS sorting or sample collections on D25 or D27.

### FACS

For single cell suspension, cells were washed with PBS and incubated with Accutase for 5 min at 37°C, three volumes of 10% FBS (Life Technologies) in PBS was added, and then centrifuged for 3 min at 200 ***g***. Cells were resuspended in FACS buffer [10% FBS, 2 µM EDTA, 0.625 mM HEPES buffer (Sigma-Aldrich) and 10 µM ROCK inhibitor in HBSS (Life Technologies)] at ∼1.5-2 million cells/ml, and transferred to 5 ml Falcon tube with a cell strainer snap cap (Falcon). FACS sorting was performed using and SH800 cell sorter (Sony Biotech), and TdTomato-expressing cells were collected into Eppendorf tubes. At least 2000 cells were collected per sample for RNA isolation.

### qPCR

Total RNA was extracted using RNAqueous micro Total RNA Isolation kit (Thermo Fisher Scientific). mRNA was reverse transcribed using iScript™ cDNA Synthesis Kit (Bio-Rad, 170-8891). Real-time quantitative PCR was performed with cDNA using HOT FIREPol^®^ EvaGreen^®^ qPCR Mix Plus (Solis BioDyne) and LightCycler^®^ 480 (Roche) for 45 cycles of 95°C for 15 s, 60°C for 20 s and 72°C for 20 s. mRNA expression was normalized to cyclophilin G (PPIG) and all the primers used are listed in Table S5.

### Immunocytochemistry

Cells were plated and grown on Geltrex-coated glass slides until fixation. First, cells were pre-fixed with 4% paraformaldehyde (PFA) (∼1:1 ratio) into cell culture medium for 5 min, washed with PBS, treated with 4% PFA for 17 min and then washed 3-4 times in PBS. For staining, cells were permeabilized with 0.05% Triton X-100 (Sigma-Aldrich) in PBS for 7 min and blocked with Ultra Vision Protein Block (Thermo Fisher Scientific). Primary antibodies used were: rabbit polyclonal anti-ISL1 antibody (Abcam, ab20670, 1:500) and guinea pig anti-GnRH antibody (EH#1018, 1:8000; a generous gift from Dr Erik Hrabovszky, Laboratory of Endocrine Neurobiology, Institute of Experimental Medicine, Hungarian Academy of Sciences, Budapest, Hungary; Hrabovszky et al., 2011; Skrapits et al., 2015). Primary antibodies were diluted in, and washed with, 0.1% Tween (Sigma-Aldrich) in PBS, and then incubated overnight at 4°C. Secondary antibodies used were: Alexa Fluor 488 donkey anti-rabbit (Life Technologies, 1:500, A21206) and Antibody-CF™594 donkey anti-guinea pig (Sigma-Aldrich, 1:1000, SAB4600096). Secondary antibodies were incubated for 35 min at room temperature, DAPI (Sigma-Aldrich) stained for 1 min, and slides were mounted with ProLong Diamond Antifade Mountant (Molecular Probes). The H9 human embryonic stem cell line (WiCell), without *GNRH1*-TdTomato reporter, was used to detect ISL1 in Fig. 3, as well as the immunocytochemistry presented in Figs S3-5, which were performed according to the same protocol. The complete list of antibodies, and their dilutions, are presented in Table S6. Images were captured using a Zeiss AxioImager.Z1 upright epifluorescence microscope and Zen Blue (Zeiss). Images were processed using ImageJ (brightness and contrast, cropping for better visibility of region of interest).

### Human tissue collection and processing

Two human fetuses (10.5 GW, post amenorrhea) were obtained with the parents’ written informed consent (Gynaecology Hospital Jeanne de Flandres, Lille, France). Permission to use non-pathological human fetal tissues was obtained from the French Agency for Biomedical Research (Agence de la Biomédecine, Saint-Denis la Plaine, France, protocol no. PFS16-002). Tissues were made available in accordance with the French bylaw (Good practice concerning the conservation, transformation and transportation of human tissue to be used therapeutically, published on December 29, 1998).

### Immunohistochemistry on human tissues

Human fetuses (10.5 weeks post amenorrhea, *n*=2) were immersion-fixed in 4% PFA in 0.1 M PBS (pH 7.4) for 1 week at 4°C, cryoprotected in 30% sucrose in PBS for 48 h, embedded in Tissue Tek (Miles) and frozen in liquid nitrogen. Tissues were cryosectioned at 18 μm using a CM3050 Leica Cryostat. Localization of ISL1 and GnRH was accomplished by using immunofluorescence procedures as previously described (Casoni et al., 2016). Briefly, sections were blocked in an incubation solution containing PBS (pH 7.4) and 0.3% Triton X-100 (TBS-T; Sigma-Aldrich, T8787) with 10% normal donkey serum (NDS; D9663; Sigma-Aldrich) for 2 h at room temperature. After blocking, sections were incubated for 48 h at 4°C with primary antibodies in a solution containing 10% normal donkey serum and 0.3% Triton X100 for 3 days at 4°C. Primary antibodies used were: rabbit polyclonal anti-ISL1 antibody (Abcam, ab20670, 1:500), guinea pig anti-GnRH antibody (EH#1018, 1:8000; a generous gift from Dr Erik Hrabovszky), previously characterized in embryonic and post-mortem human hypothalami (Casoni et al., 2016; Hrabovszky et al., 2011), and anti-TAG1 (made in goat, R&D Systems, AF2215, 1:500); 3×5 min washes in 0.01 M PBS were followed by incubation in appropriately conjugated secondary antibodies (dilution 1:400): anti-rabbit 568 (made in donkey, Invitrogen, A10042); anti-guinea pig 488 (made in donkey, Jackson ImmunoResearch, 706-545-148); anti-goat 647 (made in donkey, Invitrogen, A21082), for 1 h before incubation with Hoechst 1:1000. After 3×5 min washes in 0.01 M PBS, sections were coverslipped using lab-made (in-house) anti-fade mounting medium.

Sections were examined using an AxioImager Z1 ApoTome microscope (Carl Zeiss) equipped with a motorized stage and an OrcaFlash4.0 CMOS camera (Hamamatsu). For confocal observation and analyses, an inverted laser scanning Axio observer microscope (LSM 710, Zeiss) with an EC Plan NeoFluorÅ∼100/1.4 numerical aperture oil-immersion objective (Zeiss) was used (Imaging Core Facility of IFR114, of the University of Lille, France).

### RNA-seq

For RNA-seq, total RNA was extracted using RNAqueous micro Total RNA Isolation kit (Thermo Fisher Scientific). RNA was analyzed for concentration, integrity and quality using Qubit Fluorometer and TapeStation 4200 (Agilent). Sequencing runs were performed using the Illumina NextSeq500 sequencer (Illumina) with New England Biolabs Next Ultra Directional polyA capture method as paired-end sequencing for a read length of 75 bp. The first run contained D25 FACS-sorted TdTomato±samples (*n*=3). The second run contained D20 FGF8-treated, D20 without FGF8 treatment, and D27 TdTomato positive and negative groups (*n*=4/group). The D27 TdTomato negative samples were excluded from these comparisons, as it is possible that some GnRH neurons remained in these samples after FACS, which would lead to skewed results. RNA-seq and basic bioinformatics were performed at the Functional Genomics Unit (FUGU), University of Helsinki, Finland.

### RNA-seq data analyses

The quality of raw sequencing data in FASTQ format was analyzed using FASTQC (Simon Andrews, Babraham Bioinformatics, Cambridge, UK) and quality trimming was performed using Trimmomatic (Bolger et al., 2014). Reads were then aligned against GENCODE GRCh38 reference (Harrow et al., 2012) using STAR (Dobin et al., 2013). Quality assessment was performed using Qualimap (García-Alcalde et al., 2012) and read counts from bam files were generated using FeatureCounts (Liao et al., 2013) and annotated with ensemble release 87 using BioMart package in R (Yates et al., 2016; Durinck et al., 2009). From the raw counts, normalization and differential expression analysis were performed using DESeq package in R (Anders and Huber, 2010). We filtered all the differentially expressed genes, compared to the control, with absolute log fold change greater than 1 and less than the *P*-value of 0.05 (Benjamini-Hochberg method). In the list of differentially expressed genes, all the upregulated and downregulated genes (based on their log fold values) were considered separately for downstream analysis. Over-representation analysis was performed using Genetrail 2.0 (Stöckel et al., 2016). Bar charts representing modified log fold change of differentially expressed genes were produced using Graphpad Prism7. Gene interaction pathways were drawn using IPA (Qiagen). (https://www.qiagenbioinformatics.com/products/ingenuity-pathway-analysis).

## Supplementary Material

Supplementary information
