# Supplementary material for: Characterization of the human GnRH neuron developmental transcriptome using a GNRH1-TdTomato reporter line in human pluripotent stem cells
Source: Dis Model Mech. 2020 Mar 13;13(3):dmm040105. doi: 10.1242/dmm.040105 (PMC7075073; doi:10.1242/dmm.040105)
Supplement: Supplementary information [file dmm-13-040105-s1.pdf]

Supplementary Information

Figure S1

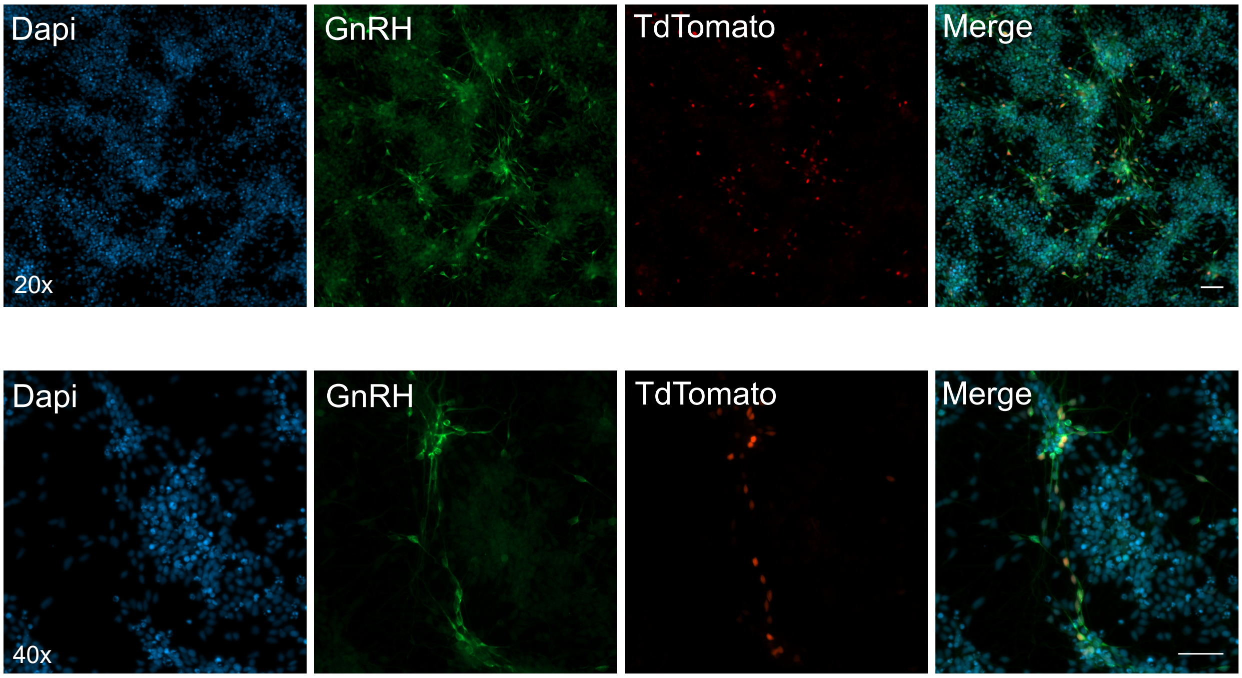

Figure S1. Validation of the H9 hESC reporter clone by immunocytochemistry.

The GnRH-TdTomato reporter generation was repeated in H9 hESCs. The sucessful clone "C11" was immunostained with anti-GnRH (Sheep) after differentiation to GnRH neurons. At day 25 of differentiation, anti-GnRH coincides with TdTomato. Scale bars indicate 50 μm. Related to Figure 1A-D.

Figure S2

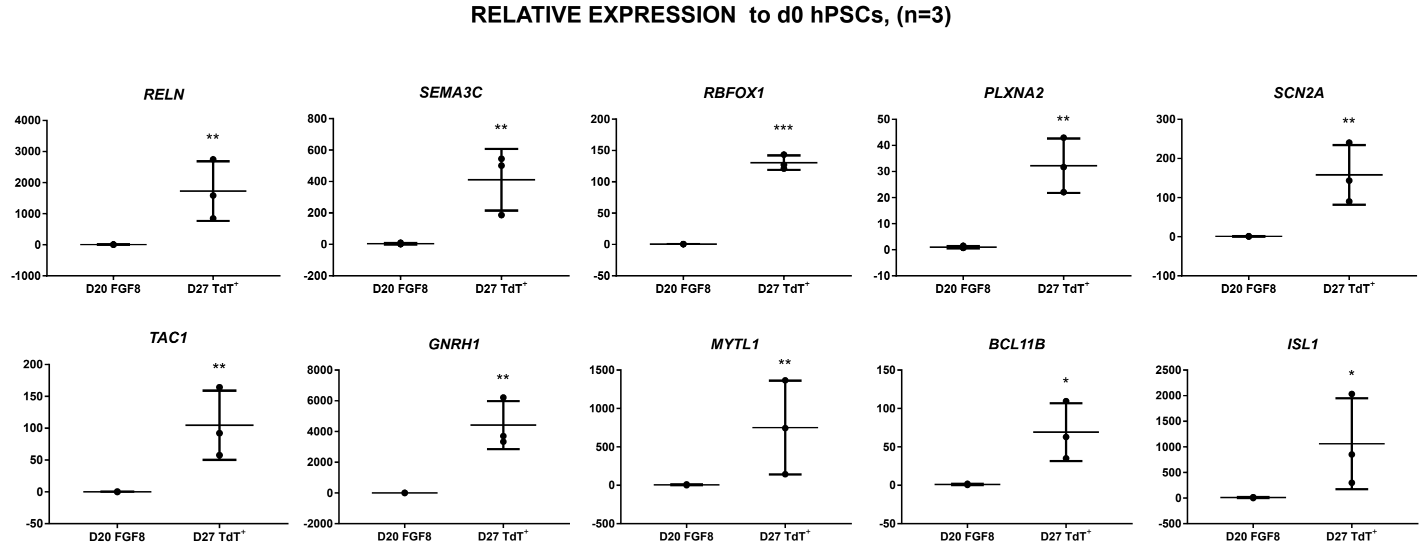

Figure S2. Validation of the RNA sequencing results by qPCR.
We performed qPCR of 10 genes that were within the top 50 most upregulated genes in Figure 2 C. Samples from D20 FGF8 neuronal progenitors, and D27 TdT+ neurons were collected from 3 independent experiments. *RELN, SEMA3C, RBFOX1, PLXNA2, SCN2A, TAC1*, and *GNRH1*, and transcription factors *MYT1L, BCL11B*, and *ISL1* exhibited increased relative expression in D27 TdT+ neurons, in accordance with the results in differential expression analysis 1. Statistical significance (ratio paired t-test) indicated as * (P≤0.05), ** (P≤0.01), and *** (P≤0.001). Related to Figure 2 C.

Disease Models & Mechanisms • Supplementary information

Figure S3

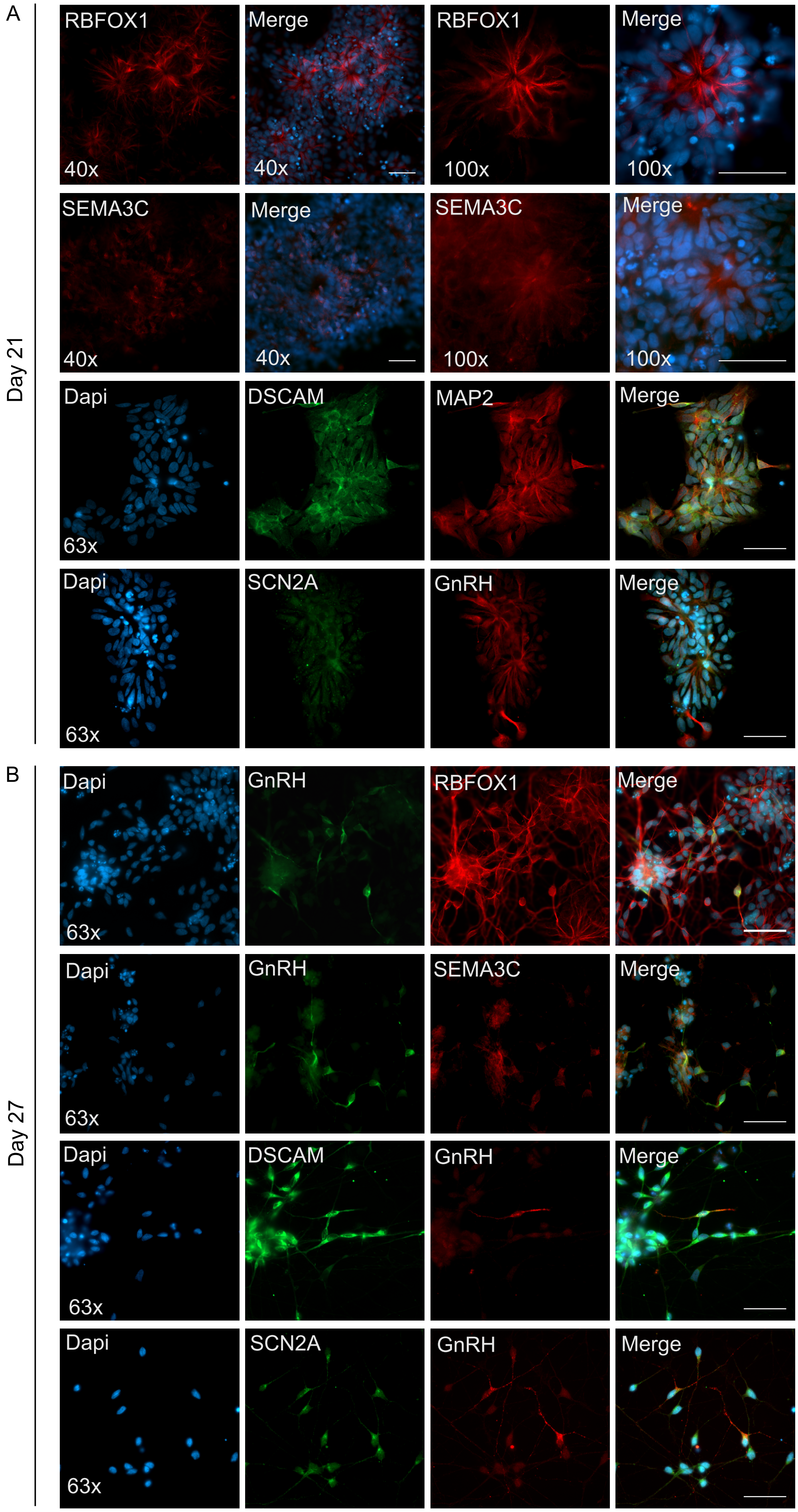

Figure S3. Immunocytochemical validation of differentially expressed genes. Immunocytochemistry at D21 of anti-RBFOX1 and -SEMA3C with nuclear staining by Dapi using 40x and 100x objectives (A, two top rows). DSCAM and SCN2A were stained together with neuronal specific anti-MAP2 and imaged using 63x objective (A, bottom rows). At D27, differentiated neuronal cultures were stained together with anti-GnRH (B). These stainings showed more abundant signal for RBFOX1, SEMA3C, DSCAM and SCN2A. Scale bars indicate 50 µm.

Disease Models & Mechanisms • Supplementary information

Figure S4

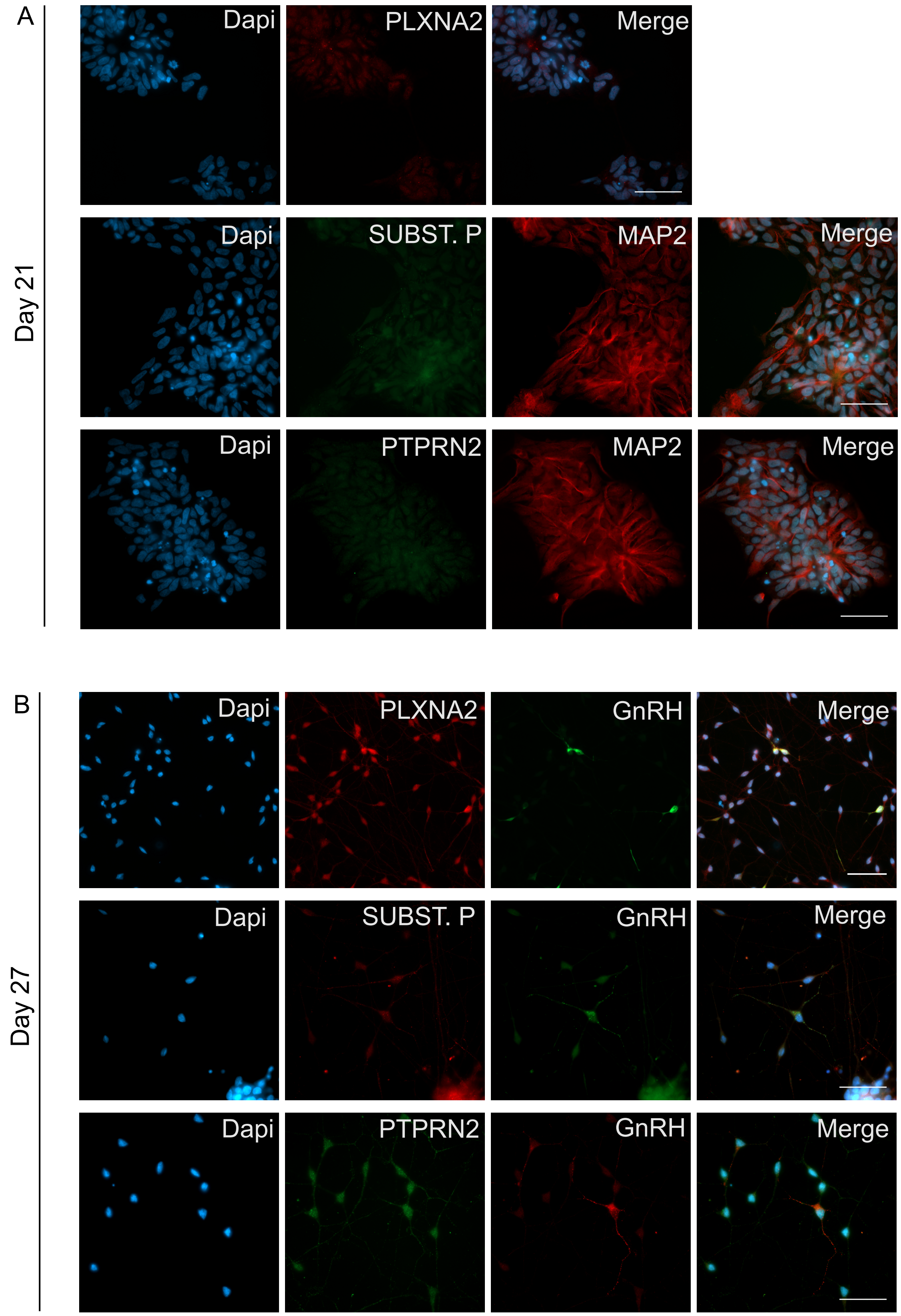

Figure S4. Immunocytochemical validation of differentially expressed genes.
Continuation of validation as described in Figure S3. Stainings were performed with antibodies for PLXNA2, Substance P (TAC1 isoform), and PTPRN2 (mature form). At day 21 Substance P and PTPRN2 were stained together with neuronal specific anti-MAP2 (A). At day 27 PLXNA2, Substance P, and PTPRN2 were stained together with anti-GnRH (B). Scale bars indicate 50 µm.

Figure S5

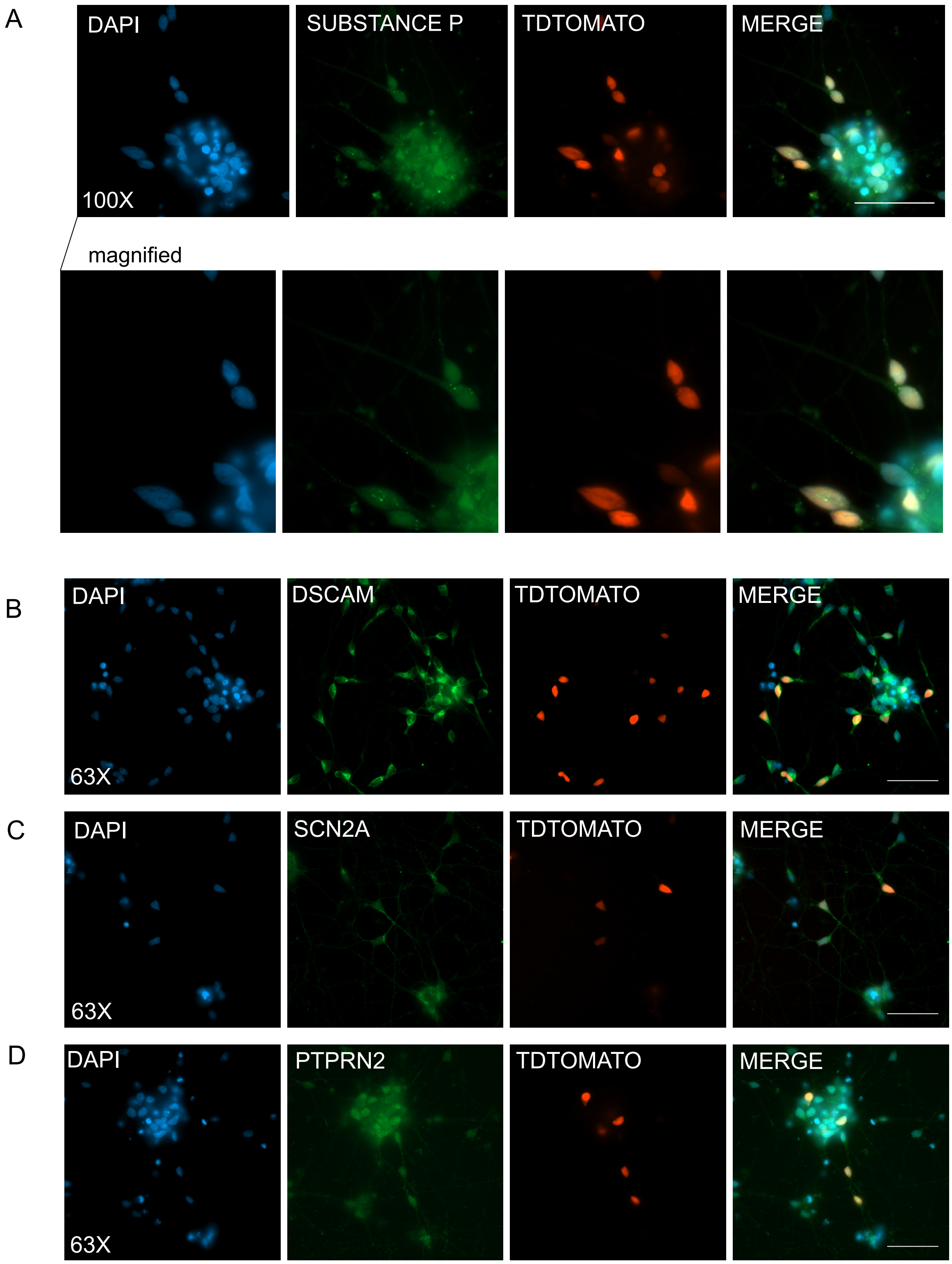

Figure S5. Immunocytochemical validation of differentially expressed genes in the GnRH-TdTomato reporter cell line.

For additional validation, we performed immunocytochemistry of Substance P (A), DSCAM (B) SCN2A (C), and PTPRN2 (D) also in the reporter cell line (TdTomato) at day 27. Stainings have been repeated at least once (N=2 (day 21) and N=3 (day 27)). Scale bars indicate 50 μm.

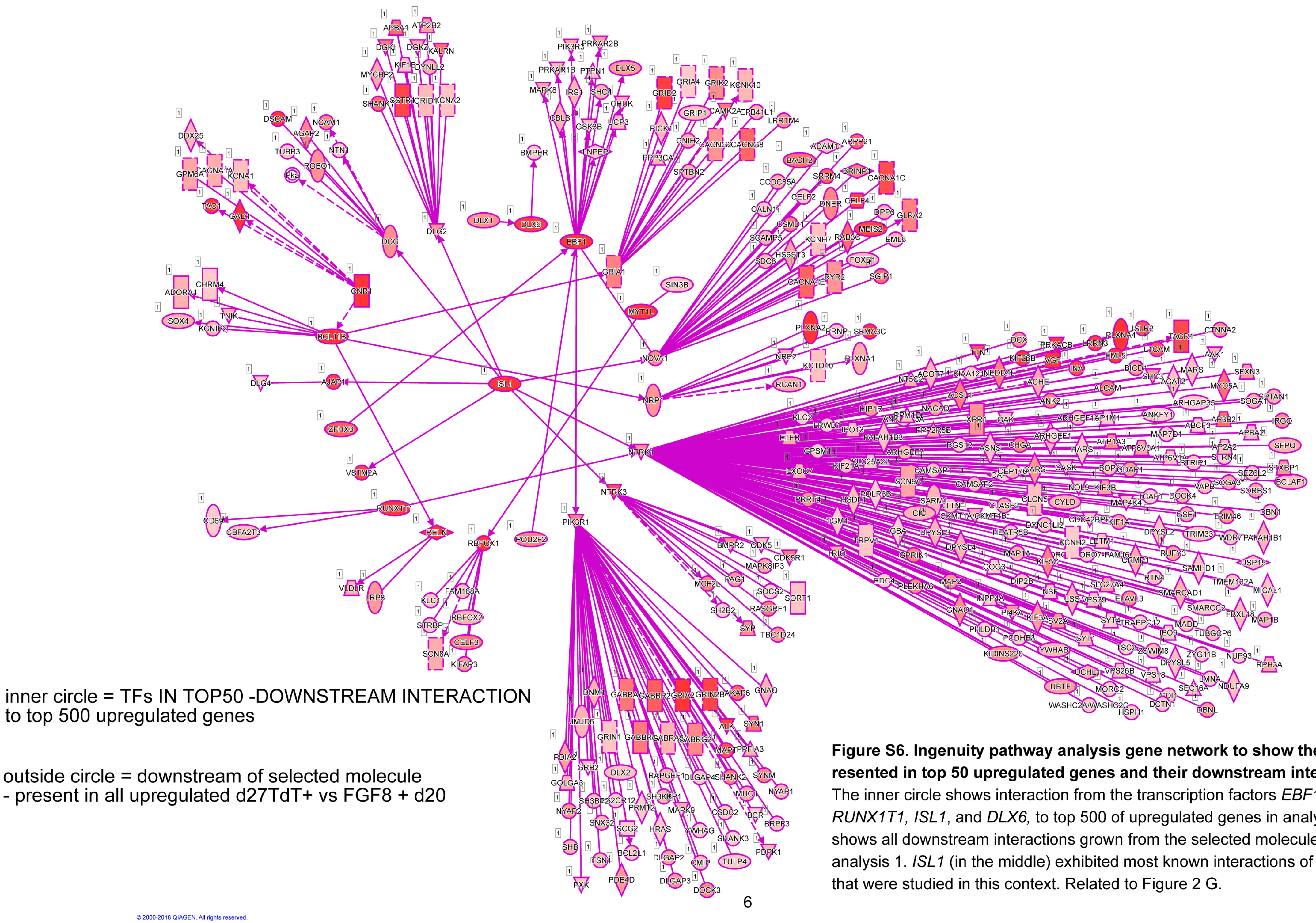

Figure S6. Ingenuity pathway analysis gene network to show the transcription factors represented in top 50 upregulated genes and their downstream interactions within the data. The inner circle shows interaction from the transcription factors *EBF1, MYTL1, BCL11B, RUNX1T1, ISL1*, and *DLX6,* to top 500 of upregulated genes in analysis 1. The outer circle shows all downstream interactions grown from the selected molecules that are represented in analysis 1. *ISL1* (in the middle) exhibited most known interactions of the 6 transcription factors that were studied in this context. Related to Figure 2 G.

Figure S7

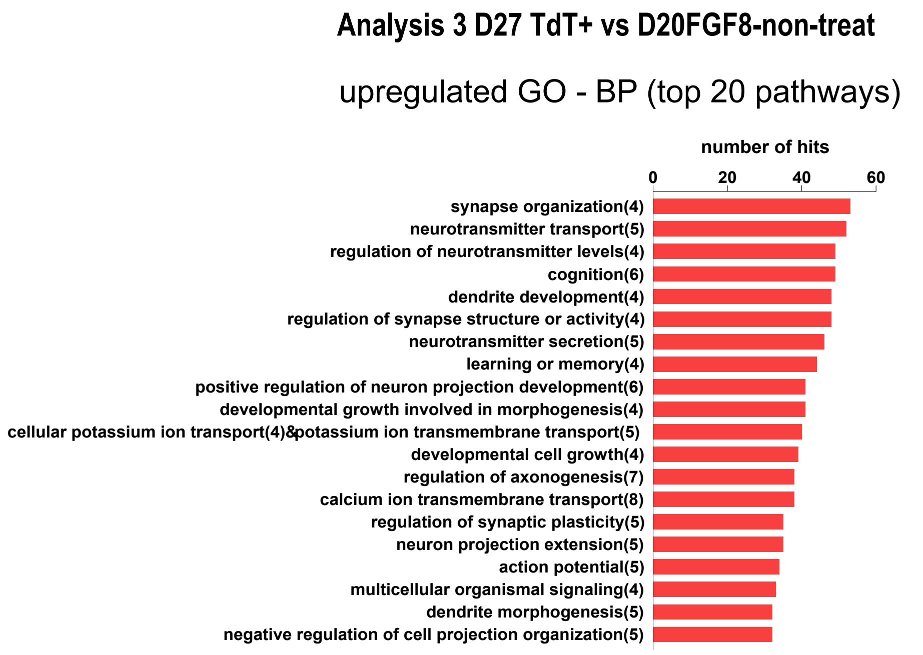

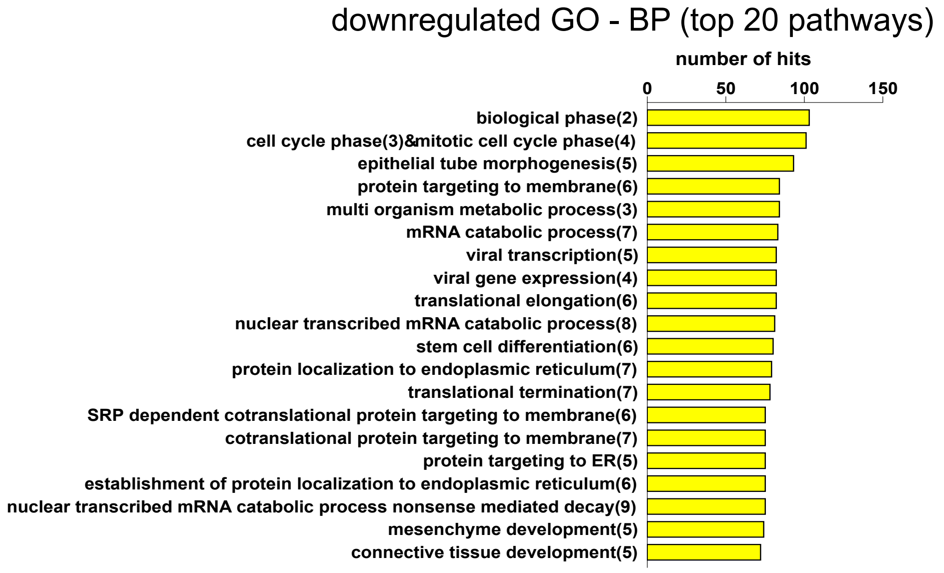

Figure S7. Over-representation analysis of differentially expressed genes in D27TdT+ vs D20FGF8nt. The terms represent 'GO - Biological processes' that were over-represented in the upregulated (upper panel) and downregulated (lower genes) genes in analysis 3. 'Upregulated' refers to higher expression in TdTomato + cells, and 'downregulated' the opposite. The top 20 significantly over-represented pathways are shown (p-value <0.005). Number of hits = number of genes per pathway that are represented in the gene lists. Related to Figure 5, Analysis 3.

Disease Models & Mechanisms • Supplementary information

Table S1. Differentially expressed genes at D25 between TdTomato-positive and -negative cell pool. Related to Figure 1F.

Click here to Download Table S1

Table S2. Top 50 upregulated and downregulated genes (D27TdT vs D20FGF8). Related to Figure 2 C.

Click here to Download Table S2

Table S3. Puberty and GnRH neuron development-associated genes that have been described in previous publications.

Genes differentially expressed (in top 50 up-/downregulated genes, Figure 2 C), reported in association with human puberty timing/development (‘Puberty’), or GnRH neurons / OP (‘GnRH neurons’) in human or animal studies. See Supplementary references.

UPREGULATED↑	PUBERTY	GNRH NEURON
RELN	Lintas and Persico, 2010 (1)	-
TAC1	Simalvi et al., 2015 (2,)	Maguire et al., 2017 (3)
GRIA2	-	Vastagh et al., 2016 (4)
GNRH1	Day et al., 2017 (5,)	Wray et al., 1989 (6)
EBF1	-	Chung et al., 2008, Kramer et al., 2000 (7, 8)
SEMA6D	Day et al., 2017 (5)	-
BCL11B	Day et al., 2017 (5)	-
PRKACB	-	Vastagh et al., 2016 (4)
GAD2	Witchel et al., 2009, Kokay et al., 2011 (9, 10)	Kokay et al., 2011 (10)
KCNMA1	-	Nishimura et al., 2008 (11)
GAD1	-	(10)
ISL1	-	Burger et al., 2018, Aguillon et al., 2018 (12, 13)
SLC35F4	-	Burger et al., 2018 (12)
VGF	Pinilla et al., 2011 (14)	-
PTPRN	Yang et al., 2018 (15)	-

Disease Models & Mechanisms • Supplementary information

DOWNREGULATED↓	PUBERTY	GNRH NEURON
NOTCH1	Quaynor et al., 2016 (16)	-
SOX3	Kim et al., 2018 (17)	-
ASPM	Passemard et al., 2009 (18)	-
AXL	Salian-Mehta et al., 2014 (19, 20)	Salian-Mehta et al., 2013 (20)
GLI3	Burger et al., 2018 (12), Quaynor et al., 2016 (16)	Burger et al., 2018 (12)
HMGA2	Zhu et al., 2010 (21)	Burger et al., 2018 (12)
SOX2	Jayakody et al., 2012 (22)	-
SPARC	-	Provenzano et al.,2010 (23)

Table S4. References from which all the pathway findings were collected (IPA ,Qiagen). Related to Figure 2 E, F, and G.

Click here to Download Table S4

Table S5. Primers used for qPCR presented in Figure S2. Related to Figure 2C.

Gene		Primers (5'-3')
RELN	Forward:	AGTGTCAGCTTGGAATTTTCTACC
	Reverse:	GGTCCAGCACAGATCTCAGG
SEMA3C	Forward:	AATCTGGAAAAGGACGCTGC
	Reverse:	TCAGTTCTGACCGCATTCCT
RBFOX1	Forward:	ATGCCACAGCACGTGTAATGA
	Reverse:	AGACTGCACCCACAACTGGATT
PLXNA2	Forward:	GCAGGCAGACAGGCACAGCA
	Reverse:	ACAGAGAGGCAGGCGTCCGT
SCN2A	Forward:	GGTTTTATTGTGAGCCTTAG
	Reverse:	CTTGAAAACTCGGAGCAGCCG
TAC1	Forward:	GCGACCAGATCAAGGAGGAAC
	Reverse:	AAAGAACTGCTGAGGCTTGGG
GNRH1	Forward:	TGCCCAGTTTCCTCTTCAAT
	Reverse:	GTCAACTGGCAGAAACCCAA
ISL1	Forward:	GCGGAGTGTAATCAGTATTTGGA
	Reverse:	GCATTTGATCCCGTACAACCT
BCL11B	Forward:	TCTCGGGTGACGGGACTCA
	Reverse:	AAGGGCTGCTTGCATGTTGTG
MYT1L	Forward:	AGAGAGCAAGTGTCCAACCC
	Reverse:	CTGTTGCTGTTGACATGCCC

Disease Models & Mechanisms • Supplementary information

Table S6. Antibodies used in Figure S3, 4, and 5. Related to Figure 2 C.

Name	ANTIBODY	CAT NO.	COMPANY	DILUTION
RBFOX1	Mouse recombinant A2BP1	NBP2-13169SS	NOVUS BIOLOGICALS	1:125
SEMA3C	Human/Mouse Semaphorin 3C Antibody(RAT)	MAB1728	R&D SYSTEMS	1:500
PLXNA2	Anti-Plexin A2 antibody (RABBIT)	ab39357	Abcam	1:500
SCN2A	Anti-SCN2A (Na_v1.2) (RABBIT)	ASC-002	alomone labs	1:200
PTPRN2	Anti-PTPRN2 (RABBIT)	HPA006900	Atlas antibodies	1:300
SUBSTANCE P *(TAC1)*	Anti-Substance P (GUINEA PIG)	ab10353	Abcam	1:200
DSCAM	Anti-DSCAM (RABBIT)	HPA0193224	Atlas antibodies	
MAP2	Anti-MAP2 (CHICKEN)	CH22103	NEUROMICS	1:1000
GNRH	Anti-GnRH (SHEEP)	#2000	gift from Prof. Erik Hrabovzky (PMID:25713511)	1:4000
GNRH	Anti-GnRH (GUINEA PIG)	#1018	gift from Prof. Erik Hrabovzky(PMID: 22654828)	1:16000
**Secondary antibody**				
Donkey-anti-sheep	Antibody-CF™488	SAB4600038	SIGMA	1:1000
Donkey-anti-guine pig	Antibody-CF™594	SAB4600096	SIGMA	1:1000
Donkey-anti-guine pig	Antibody-CF™488	SAB4600033	SIGMA	1:1000
Donkey-anti-mouse	Alexa Fluor 594	A21203	Invitrogen by Thermo Fisher Scientific	1:500
Goat-anti-chicken	Alexa Fluor 594	A11042	Invitrogen by Thermo Fisher Scientific	1:500
Donkey-anti-rabbit	Alexa Fluor 594	A21207	Invitrogen by Thermo Fisher Scientific	1:500
Donkey-anti-rabbit	Alexa Fluor 488	A21206	Invitrogen by Thermo Fisher Scientific	1:500
Donkey anti-rat	Alexa Fluor 594	A21209	Invitrogen by Thermo Fisher Scientific	1:500

Table S7. All overlapping genes upregulated or downregulated in both analyses 2 and 3. Related to Figure 5.

Click here to Download Table S7

Table S8. References from which all the pathway findings were collected (IPA ,Qiagen). Related to Figures 5 D, and E.

Click here to Download Table S8

Disease Models & Mechanisms • Supplementary information

Supplementary Materials and Methods

The background information on how the genome editing to generate a reporter into the *GNRH1* gene of human pluripotent stem cells was planned. Related to Figure 1 and Materials and Methods in the main text.

1. Design of GNRH1 reporter cell line, genome editing by CRISPR-Cas9

GNRH1 Genomic sequence:

Homo sapiens gonadotropin-releasing hormone 1 (luteinizing-releasing hormone) (GNRH1), RefSeqGene on chromosome 8

NCBI Reference Sequence: NG_016457.1

GenBank Graphics

>gi|285026423:5001-10783 Homo sapiens gonadotropin-releasing hormone 1 (luteinizing-releasing hormone) (GNRH1), RefSeqGene on chromosome 8

Bold = exon

Stop codon

AATTTCTCATCTTTCTGTTCCCACTGCCCTTAAGAGTGATTTGCATATTTAACTCAATAAGCATCTACTG
AAATGAGTTGATCTGTTGATGTAAGTCTGCTCAATATGGTCTTGCTCTCAGAATATGTTTCTTGCCTTTT
TGATGCTTTAGAAGGCTTTCAAGGTAAGTCAAGCAGGGAACCTGGTGGGTAGATGAGGGAATTTTCAAAC
ACACAACTGTCTGATTTAGGATCCTACATGGACTTGGTATATAGTGTCACTTACTTGTAAATCAGATTTT
TAAAATTGGAAGCAACTCTGTGATCATCTAGTCCATCTAGTCTACACCCTTCCTTTTACAAATGAAGAAT
CCAAGAGCCAGAAGCTCCCAGACATCCTGACTCAATGTCCTATATTTGTTGTATAGCCTCCTTTGTGGAA
GTTATGTATGCATTTGACTTCACTTAATCTAAGACATCTATTTTCCTTGAACTCTTGATAGGTCTGCTGG
TTTCCTCAAGGGAATCCAATCTAGCTGGATTTTAATCTCTTTGAATTGTGTCCTCAGCTATAAAAGTTTT
AGCTGAGGTTTTAATGGCTGCACTTAAGTAAATCTAACAGATATACCAGGGGGTGTTCCAATTACATACA
CCATTAAAGGGCTTTATGTGAGGATTTTTAAAAATTACCATTAAAAAAAAAAAAAGCATAGTCCATTTGCA
GTATAATTTACCAGCAGGAAAGATTTCAATGTCCTGGAAAAATTCCCTATAAAAAGGAAGATAGGAAAAC
AGAAAAGTCACAGTACTCAACCTACTTCAAGGGAAGATTGGGATCTTTTTGGCTCTCTGCCTCTAAACAG
GTAAAAGGCTTTGTATTATTTCTAGCACGAGTTTTTCTTCTTTAGATTGCATGCTATTGTATGTCTACAG
GGCATTTGACAGCCCAAGGGCTAAATCCAGGTGTGACGGTATCTAATGATGTCCTGTCCTTCACTGTCCT
TGCCATCACCAGCCACAGAGATCCAGGCTTTGGGGACTCCCACAGCTTATCGACCAGTGTTTGATTTAGT
TTTTAGCCTCTTTCCCATCAAATGAAAATTAACTTGGAGACACATTTCATTAGAAAATTAGAGGCCCCCT
TGGCTAGGAAGGCATCTGGTCTGGGACTAACTACTTTGAACAGTGTTGAGTCCTCTCTCCCACAGATGGT
TCAGCCAGCAGTAATGCTAGGAAGACTGAAGGATAAATAGAAAAATGTCATTAGTACCATGGGGTAGCCA
TGTAATGTCAAGCAATTTTATATTAGCCAGAGATTCCTAGTAGGAGCTACTTTTCTTAACAGATGACTCA
GTTCTCTTTATCTCAGGAATGAAAGAGTTGAAGACCAATCCACAACAGGGGAAATGTTAAGGCAAAATGA
TGAACTTGATAAGGGATGAATTATGGGGTTTGGATAACCAAACAATAAAAATAAAAGTATAGACTATTTT
AGTACTAAAAAGGTCCTGAACATGTGAGCTTAAGTACTCATTTTGTCCCCAGTGGCTAAGAAACTAAAGG
CAAGCCAGCAAGTGTCTCTGAGTTTCAGTGTCTGTATGTAAAAACTGACTCTGACTTCCATCTTCTGCAG
GGTTAGTGATACAGATGCTAGCTTTTTCACTAAAGAGGTCTTTTAGTTTATACTCAACCTTGTCTGGATC
TAATTTGATTGTGCATTCATGTGCCTTAGAATGAAGCCAATTCAAAAACTCCTAGCTGGCCTTATTCTAC
TGACTTGGTGCGTGGAAGGCTGCTCCAGCCAGCACTGGTCCTATGGACTGCGCCCTGGAGGAAAGAGAGA
TGCCGAAAATTTGATTGATTCTTTCCAAGAGGTAAGTTTCTCTCAGCTTCAAAATAAGACATAGTGATTT
TATTCAATTTAACTATATTAAACATTCAGGATAGCCCCAATGTCAATATTCTATGATGTTGTACCCTAGA
TGCTCCAGGTGAGATAAGGCACTTACAAAGTAGAAGTCCCATTCCTACTTTCAGTTCACACAGGGACTAA
ACAAAGAGCTGGAAAAATTCCAAAAGAATATATTAATAGCAACAAGTGTGAGACAGCACGTCATACTCTG
AGTGTATGGGATTGCTAAAGGAATTAGAAACAATGGGATAGGGTCAAGGTCTGTATCAGAAGATGATTCT
TTGGGATTTGGAAAAAACGTTAGAACTTCGTTTTTTTTTTCCTCATTTCATCTTTTAAACATATCTATGC
TATTAGATCAGTACATTTTTATAATTTATAAATGAGTATCCTTATCAAGGATGCATGCCCTATAATTTCT
TTTCACTGATAGGGGCATTTAAGCAAAGTTGGAGACTGGTAGAATACAGGAATTAGCAAACTCAAGATGA
TAAGATAACGTAGTAGAAAACATGCTGATTTAAATTCATATAGATTAGATTATAGGACTGGCACCAACAC
TAACGTGAGGTACTTGCTTTCTTGTTTTTTGGGGTTATTTTCTAAGACAGGGTCTCGCTGTGTTATCTAG
GCTGGAATTTAGTGGCACGATCACAGCTCACTGCAGCCTTGACTTCTTGGGCTCAAGTGATCCTCCCACC
TCAGCCGTCTGCATAGCTGGGACCACAGGCATGCACTATCACACCCAACTAATTTTTAAAACTTTTTATA
GAGATGGGGTTTCCCTATGTTGCCCAGGCTGGTCTCAAACTCCTGGGCTCAAGGGATCCTCCCACCTTGG
CCTCCCAAAGTGCCGGGAGGATTTTTGTTTATAAAATATGACCACTCATCAGGGTCATGTAAGGAAAGAA

Disease Models & Mechanisms • Supplementary information

GCCATCTATGTTAGCTGATTAACCTGAAAAATAACCTAGGACTGAGAATGGGAAAAATTTTAAATCATTT
CATTATCATTGGAAGGAATTATCTCTTTTCTGAGAAATAATAAAGATAATTTAGTATTAAAGAAGACCCA
GAATCTGAAGCCTCTTCTCTGCAGGTTATACATGAAGCAAATCTCATTGAACTATAACATATTTAGTAAA
ACCTAGAAAATAAAAACCAACCTTTTTTTACAACTATAAACTCTTGGGGGTTTTTTGCTTTTTGTTTTTT
TGGGTATACTGACTCTCATGAGGCTCAAAGTGCCTCCCTCTTTTCATCTTTAAGGGGAAAATATGATATC
TTCTTACTGTCTCCATTATCTCCAGATCCCCATGCCATTCAGTAGAAATGTCAGATGGCAGATCTGTGTC
CTTAAAGTCCCTATGCTAATCCTGCAACTTTCCCAATCTCCCCCAGCCCCACATCCCCCTTGACCCCACT
CTCCACAATTTTTTGGTGGAAATGGAAAACACCATTTCATTTCTTTATCTCCATCTCAAAGCATCACATT
CTCTTTCTTCAG**ATAGTCAAAGAGGTTGGTCAACTGGCAGAAACCCAACGCTTCGAATGCACCACGCACC**
AGCCACGTTCTCCCCTCCGAGACCTGAAAGGAGCTCTGGTAAGTTAAAGTGATCATAACATGATCACAGC
ATAGAGCTCTAGAGGTGGATAAGCCTTTGGGGATCACTTAGGACAGCTACCTCCCAGATACTGTGGGGCT
TACATTCCTGACTCCTCTGTTACCTCCTGTGGTAGGACCGTGTTTCATGACAATCCCAGTTGGTGGTTAG
ACAGCACTAGGGGCTGAAACGTTTTTTTGTTTGTTTGTTTTTTCTGAAGTTGAATTGAAATCTCTCTGTA
ACTTTTATCTCTTAATCCTGGTTCTAGCTTTTGGGATAACTAACAAAACAAATTTCTTCCCACTGCTGCA
TTTCATTTCTTCAAGTAAAATCACCAAACCCCCTAGACTACTCCACTCCAGGCTACTCCACAGCCCTCCA
CCTGATCCTGCAACTGTGCTTTATCTTACATGGTTTTCCAGAACCTTGGGGAATAGAGACATGAGAAACA
CTGCTGTAGATGGGTTTTTTTTTCTCTTCTTTGGAATGAAAAATGCCAAACTACTAAATTTATAATTTAG
AGAGTGATGGACTTGATTTCCAGTTTCCTGATAGGACAATAATCACCTCCAAATTCCGCCCCCCAAAATG
GAAATACACTAATCATATTAGGTTTTTGATGAAAAAGTATAAAGAGAATTGAATGTATAAATTGAATCTT
TTAAAAAAATTATTTGTTGAGACAGGATCTTGCTCTGTTCCCCGGGCTAGAGTGCAATGGTGCAATCAAT
GCTCACCACAGCCTCAACCTCCCAGGCTCAAGCAATTCTGGAGACTAGATTAGCCTCTCTAGTAGCTGGG
ACTATAAGCACTTGCCACCACACCCAGCTGATTTTATTTTTTAATTTTTTGTAGAGACAGGGTCTATGTT
GCCCAGGCTGGTCTCAAACTCCCAGCCTCAAGATCCTCCCAAAGTGCTGGGATTAAAGGTGCAAGCCACT
GTGCCTGACTTAAGTTGAATCTTGGATTCAATGTTGATATTCTCTGATCTCTATTGTCCACTTATCTGCA
GCAATCAGAAGGCATTACAGTTAATGATCAGTTATGCCTAGGAGCTGGGAAAGCCCAAATAAATCATATA
TAAAAATAAGCTGTAATTTTAATTGTCTACAGTGACTTCAACTTAATATACCCACAGAACAAAGAAAAAA
GTGGGCAGACGTCGTTATTTCCTTTTTCGTTTTTTTTGGAGTGCAGTGGCGCAATCTCGGCTCAATGCAA
CCTCCATCTCCTGGGTTCAAGCGATTCTCCTGACTCAGCCTCCCGAGTAGCTGAGATTACAGGCATGAGC
CACCACACTGGACTAATTTTTGTATTTTTAGTAGAGATGGGGTTTCACCATGTTGGCCAGGATGGTCTCA
ATCCCTTAACCTTGTGATCCGCCCACCTCGGCCTCCCAAAGTGCTGGGATTACAGGCATGAGCCACCACG
CCCAGCCTATTTCCTTTCTTTTTCTAATCTTGCTTACTGCATTACAAAAATGGCAAGCAGTGAAATTTGT
CAAACATGACATTATGAAGAAATTGAAGCAAAGGCTGGTTTAATAGCAAAGTAATTGACCAGACTTTTTT
T**TCACTTCCTTCCTCACAACTCATCC**TTAAACTATTAATGTAGATTTTATGTATATTAAGTGCTTAAAAA
GACCCAATCGGCCAGGCACAGTGGCTCATGCCTGTAATCCTAGCATTTTGGGAAGCCGAGGTAAGTG GAT
CACTTGAGGCCAGGAGTTCAAGACCAGCCTGGCCAACATGGTGACACCCTGTCTCTACTAAAACTATAAA
AATTAGCCAGATGCGGTGATGCATGCCTGCAATCCCAACTACTAGAGAGGCTGAGGCACGAGAATCATTT
GAACCTGGAAGGTGGAGGTTGCCATGAGCTGAGATCATACTACTGCACTCCAGCCTGGGTGACAGAGTGA
GGTTCTGTCTCAAAAAAAAAAAAAAACCAGAAAAACAAACCCAATTTATCATGTCTCCCTAGCACTAACTA
GAGCACAAAATCAAACAGACCAATTCCTTCCAGACTGATATTTTAGAAATTAAAATGTCAAAATGTAATG
AAATTCAGCTGGTAAAGTCAGTCTTGATATATTTGTTATATATTTTTCAGGAAAGTCTGATT**GAAGAGGA**
AACTGGGCAGAAGAAGATTTAAATCCA**TTGGGCC**AGAAGGAATGACCATTACTAACATGACTTAAGTATA
ATTCTGACATTGAAAATTTATAACCCATTAAATACCTGTAAATGGTATGAATTTCAGAAATCCTTACACC
AAGTTGCACATATTCCATAATAAAGTGCTGTGTTGTGAATGAAGTGGCATACCTGTTAAATCTTTCTCCAACTCAGAACTCCGGGG
GAAGGATCACTGTAAACCCACCAAAGGGAGCCCTCCATGTGTGTATACAGGTGGCAGATGGGAGGGCAGGTAAGATAAAGTGTCTG
TTGTTGACAAAAGGGATCTCAGGCTCTCCAGCACCCATACCCTGCATCTACCCACAAGCAGAACAGCCACATACTGGTCCAGCCAG
AAAAAGCTGATTCAGCTCCAGTTTCTCTGGGATTATAACTTTATCTTCGACCATACTCTTCAGAAGTTGAGGTGGGGCCACGGCCA
AGGCTTTCTTCCACTTGGAAAGAAGTTCCCTCCCTTGATCGTCTCCAAACCCTTTGAAAGTTTACTGGAACCCAAATGAGGCCTGG
GGGTAAGGAGAGGGGGCCTCCAAGGACTCCTAGTCCAGCGCTC**CTTCTGGTCCCACGCAATCT**ATCCAAGTGGTGCACACT
G AGGGTTGGGACTGAGACCTA
AAAAATAATAGAGTGTTCATCTGCTTGATCTGTTTGGTTTGCATTTAAGAAACACAATGGAGTACAGACAGACCGTTGGAGATGGG
ACTCTATTGTTCCTATGTCCCCT

2. RNA guide oligo

"Guide 3:"
5’- GTGGAAAGGACGAAACACCg **GAAGAAGATTTAAATCCATT** gttttagagctaGAAAtag
-3’

3. Homologous arms

Primers designed for H9 GDNA cloning of homologous arms:

		TM
5ha_GNRH1_BamH1_F	**gcatGGATCC**[BamH1]**TCACTTCCTTCCTCACAACTCATCC**	62.2
	**gcga GCTAGC**	TM
5ha_GNRH1_Nhel_R	**AATCTTCTTCTGCCCAGTTTCCTCTTC**<br>(GAA GAG GAA ACT GGG CAG AAG AAG ATT GCTAG C[NheI]tcgc[overhang])	63.7
	**gcat GGCGCGCC**[AscI]	TM
3ha-GnRH1-AscI-Fw:	**AGAAGGAATGACCATTACTAACATGAC**	59.2
3ha-GnRH1-XbaI-Rv:	gcatt TCTAGA AGATTGCGTGGGACCAGAAG<br>**(Reverse: CTTCTGGTCCCACGCAATCT TCTAGA**[XbaI]**)**	TM60.0

4. Primers: Detection of Donor template introduction

Design of primers for insertion detection

Primer pair 1:

Forward : **CCACGCCCAGCCTATTTCCTTT**

Reverse (compliment): **gatgacctcctcgcccttgc**

Primer pair 2:

Forward : ggtccctcgaagaggttcac

Reverse (compliment): **CGGTCTGTCTGTACTCCATTGT**

Gnrh1 sequence

Donor template integrated correctly

GCAATCAGAAGGCATTACAGTTAATGATCAGTTATGCCTAGGAGCTGGGAAAGCCCAAATAAATCATATATAAAAATAAGCTGTAATTTTAA
TTGTCTACAGTGACTTCAACTTAATATACCCACAGAACAAAGAAAAAAGTGGGCAGACGTCGTTATTTCCTTTTTCGTTTTTTTTGGAGTGC
AGTGGCGCAATCTCGGCTCAATGCAACCTCCATCTCCTGGGTTCAAGCGATTCTCCTGACTCAGCCTCCCGAGTAGCTGAGATTACAGGCAT
GAGCCACCACACTGGACTAATTTTTGTATTTTTAGTAGAGATGGGGTTTCACCATGTTGGCCAGGATGGTCTCAATCCCTTAACCTTGTGAT
CCGCCCACCTCGGCCTCCCAAAGTGCTGGGATTACAGGCATGAGCCA**CCACGCCCAGCCTATTTCCTTT**CTTTTTCTAAT
CTTGCTTACTGCATTACAAAAATGGCAAGCAGTGAAATTTGTCAAACATGACATTATGAAGAAATTGAAGCAAAGGCTGGTTTAATAGCAAA
GTAATTGACCAGACTTTTTTTTCACTTCCTTCCTCACAACTCATCCTTAAACTATTAATGTAGATTTTATGTATATTAAGTGCTT
AAAAAGACCCAATCGGCCAGGCACAGTGGCTCATGCCTGTAATCCTAGCATTTTGGGAAGCCGAGGTAAGTGGATCACT
TGAGGCCAGGAGTTCAAGACCAGCCTGGCCAACATGGTGACACCCTGTCTCTACTAAAACTATAAAAATTAGCCAGATG
CGGTGATGCATGCCTGCAATCCCAACTACTAGAGAGGCTGAGGCACGAGAATCATTTGAACCTGGAAGGTGGAGGTTG
CCATGAGCTGAGATCATACTACTGCACTCCAGCCTGGGTGACAGAGTGAGGTTCTGTCTCAAAAAAAAAAAAAACCAGAA
AAACAAACCCAATTTATCATGTCTCCCTAGCACTAACTAGAGCACAAAATCAAACAGACCAATTCCTTCCAGACTGATATT
TTAGAAATTAAAATGTCAAAATGTAATGAAATTCAGCTGGTAAAGTCAGTCTTGATATATTTGTTATATATTTTTCAGGAAA
GTCTGATTGAAGAGGAAACT**GGG**CAGAAGAAGATT_GCTAGCGTCGACggctccggagagggcagaggaagtctgctaacatgcggtgacg
tcgaggagaatcctggcccaATCGATGGTccaaaaaagaagagaaaggtaTTCGATGGTccaaaaaagaagagaaaggtaTTCGATatggtga**gca**

Disease Models & Mechanisms • Supplementary information

agggcgaggaggtcatcaaagagttcatgcgcttcaaggtgcgcatggagggctccatgaacggccacgagttcgagatcgagggcgagggcgagggc
cgcccctacgagggcacccagaccgccaagctgaaggtgaccaagggcggccccctgcccttcgcctgggacatcctgtcccccagttcatgtacggctccaaggcgtacg
tgaagcaccccgccgacatccccgattacaagaagctgtccttccccgagggcttcaagtgggagcgcgtgatgaacttcgaggacggcggtctggtgaccgtgacccagga
ctcctccctgcaggacggcacgctgatctacaaggtgaagatgcgcggcaccaacttcccccccgacggccccgtaatgcagaagaagaccatgggctgggaggcctccac
cgagcgcctgtaccccgcgacggcgtgctgaagggcgagatccaccaggccctgaagctgaaggacggcggccactacctggtggagttcaagaccatctacatggcca
agaagcccgtgcaactgcccggctactactacgtggacaccaagctggacatcacctcccacaacgaggactacaccatcgtggaacagtacgagcgctccgagggccgc
caccacctgttcctggggcatggcaccggcagcaccggcagcggcagctccggcaccgcctcctccgaggacaacaacatggccgtcatcaaagagttcatgcgcttcaag
gtgcgcatggagggctccatgaacggccacgagttcgagatcgagggcgagggcgagggccgcccctacgagggcacccagaccgccaagctgaaggtgaccaaggg
cggccccctgcccttcgcctgggacatcctgtcccccagttcatgtacggctccaaggcgtacgtgaagcaccccgccgacatccccgattacaagaagctgtccttccccga
gggcttcaagtgggagcgcgtgatgaacttcgaggacggcggtctggtgaccgtgacccaggactcctccctgcaggacggcacgctgatctacaaggtgaagatgcgcggc
accaacttcccccccgacggccccgtaatgcagaagaagaccatgggctgggaggcctccaccgagcgcctgtaccccgcgacggcgtgctgaagggcgagatccacc
aggccctgaagctgaaggacggcggccGctacctggtggagttcaagaccatctacatggccaagaagcccgtgcaactgcccggctactactacgtggacaccaagctgg
acatcacctcccacaacgaggactacaccatcgtggaacagtacgagcgctccgagggccgccaccacctgttcctgtacggcatggacgagctgtacaagtaaTTTAAA
GAATTCCGCCCCCCCCCCCTCTCCCTCCCCCCCCCCTAACGTTACTGGCCGAAGCCGCTTGGAATAAGGCCGGTGTGC
GTTTGTCTATATGTTATTTTCCACCATATTGCCGTCTTTTGGCAATGTGAGGGCCCGGAAACCTGGCCCTGTCTTCTTGA
CGAGCATTCCTAGGGGTCTTTCCCCTCTCGCCAAAGGAATGCAAGGTCTGTTGAATGTCGTGAAGGAAGCAGTTCCTCT
GGAAGCTTCTTGAAGACAAACAACGTCTGTAGCGACCCTTTGCAGGCAGCGGAACCCCCCACCTGGCGACAGGTGCCT
CTGCGGCCAAAAGCCACGTGTATAAGATACACCTGCAAAGGCGGCACAACCCCAGTGCCACGTTGTGAGTTGGATAGTT
GTGGAAAGAGTCAAATGGCTCTCCTCAAGCGTATTCAACAAGGGGCTGAAGGATGCCCAGAAGGTACCCCATTGTATGG
GATCTGATCTGGGGCCTCGGTGCACATGCTTTACATGTGTTTAGTCGAGGTTAAAAAAACGTCTAGGCCCCCCGAACCA
CGGGGACGTGGTTTTCCTTTGAAAAACACGATGATAATATGGCCACAACCATGACCGAGTACAAGCCCACGGTGCGCCT
CGCCACCCGCGACGACGTCCCCAGGGCCGTACGCACCCTCGCCGCCGCGTTCGCCGACTACCCCGCCACGCGCCACA
CCGTCGATCCGGACCGCCACATCGAGCGGGTCACCGAGCTGCAAGAACTCTTCCTCACGCGCGTCGGGCTCGACATC
GGCAAGGTGTGGGTCGCGGACGACGGCGCCGCGGTGGCGGTCTGGACCACGCCGGAGAGCGTCGAAGCGGGGGCG
GTGTTCGCCGAGATCGGCCCGCGCATGGCCGAGTTGAGCGGTTCCCGGCTGGCCGCGCAGCAACAGATGGAAGGCCT
CCTGGCGCCGCACCGGCCCAAGGAGCCCGCGTGGTTCCTGGCCACCGTCGGCGTCTCGCCCGACCACCAGGGCAAG
GGTCTGGGCAGCGCCGTCGTGCTCCCCGGAGTGGAGGCGGCCGAGCGCGCCGGGGTGCCCGCCTTCCTGGAGACCT
CCGCGCCCCGCAACCTCCCCTTCTACGAGCGGCTCGGCTTCACCGTCACCGCCGACGTCGAGGTGCCCGAAGGACCG
CGCACCTGGTGCATGACCCGCAAGCCCGGTGCCTGACGCCCGCCCCACGACCCGCAGCGCCCGACCGAAAGGAGCG
CACGACCCCATGCATcgaaGATCTAGAGCTCGCTGATCAGCCTCGACTGTGCCTTCTAGTTGCCAGCCATCTGTTGTTTG
CCCCTCCCCCGTGCCTTCCTTGACCCTGGAAGGTGCCACTCCCACTGTCCTTTCCTAATAAAATGAGGAAATTGCATCG
CATTGTCTGAGTAGGTGTCATTCTATTCTGGGGGGTGGGGTGGGGCAGGACAGCAAGGGGGAGGATTGGGAAGACAAT
AGCAGGCATGCTGGGGATGCGGTGGGCTCTATGGCTTCTGAGGCGGAAAGAACCTGCAGCCCAAGCTTGGCGTAATCA
TGGTCATAGCTGTTTCCTGTGTGAAATTGTTATCCGCTCACAATTCCACACAACATACGAGCCGGcAATTCcgatcatattcaata
acccttaatataacttcgtataatgtatgctatacgaagttattaggtctgaagaggagtttacgtccagccaagcttaggatctcgacctcgaaattctaccgggtaggggaggcgc
ttttcccaaggcagtctggagcatgcgctttagcagccccgctggcacttggcgctacacaagtggcctctggcctcgcacacattccacatccaccggtagcgccaaccggctc
cgttctttggtggccccttcgcgccaccttctactcctcccctagtcaggaagttcccccccgccccgcagctcgcgtcgtgcaggacgtgacaaatggaagtagcacgtctcacta
gtctcgtgcagatggacagcaccgctgagcaatggaagcgggtaggcctttggggcagcggccaatagcagctttgctccttcgctttctgggctcagcagctgggaagggtgg
gtccgggggcgggctcaggggcgggctcaggggcggggcgggcgcccgaaggtcctccggaggcccggcattctgcacgcttcaaaagcgcacgtctgccgcgctgttctc
ctcttcctcatctccgggcctttcgacctgcatccatctagatctcgagcagctgaagcttaccatgaccgagtacaagcccacggtgcgcctcgccacccgcgacgacgtcccc
agggccgtacgcaccctcgccgccgcgttcgccgactaccccgccacgcgccacaccgtcgatccggaccgccacatcgagcgggtcaccgagctgcaagaactcttcctc
acgcgcgtcgggctcgacatcggcaaggtgtgggtcgcggacgacggcgccgcggtggcggtctggaccacgccggagagcgtcgaagcgggggcggtgttcgccgaga
tcggcccgcgcatggccgagttgagcggttcccggctggccgcgcagcaacagatggaaggcctcctggcgccgcaccggcccaaggagcccgcgtggttcctggccacc
gtcggcgtctcgcccgaccaccagggcaagggtctgggcagcgccgtcgtgctccccggagtggaggcggccgagcgcgccggggtgcccgccttcctggagacctccgc
gccccgcaacctccccttctacgagcggctcggcttcaccgtcaccgccgacgtcgaggtgcccgaaggaccgcgcacctggtgcatgacccgcaagcccggtgcctgacg
cccgccccacgacccgcagcgcccgaccgaaaggagcgcacgaccccatgcatcgatgatatcagatccccgggatgcagaaattgatgatctattaaacaataaagatgt
ccactaaaatggaagtttttcctgtcatactttgttaagaagggtgagaacagagtacctacattttgaatggaaggattggagctacgggggtgggggtggggtgggattagata
aatgcctgctctttactgaaggctctttactattgctttatgataatgtttcatagttggatatcataatttaaacaagcaaaaccaaattaagggccagctcattcctcccactcatgatct
atagatctatagatctctcgtgggatcattgtttttctcttgattcccactttgtggttctaagtactgtggtttccaaatgtgtcagtttcatagcctgaagaacgagatcagcagcctctgtt
ccacatacacttcattctcagtattgttttgccaagttctaattccatcagaagctggtcgagatccggaacccttaatataacttcgtataatgtatgctatacgaagttattaggtc
cctcgaagaggttcactaGGCGCG_CCAGAAGGAATGACCATTACTAACATGACTTAAGTATAATTCTGACATTGAA
AATTTATAACCCATTAAATACCTGTAAATGGTATGAATTTCAGAAATCCTTACACCAAGTTGCACATATTCCATAATAAAGT
GCTGTGTTGTGAATGAAGTGGCATACCTGTTAAATCTTTCTCCAACTCAGAACTCCGGGGGAAGGATCACTGTAAACCCA
CCAAAGGGAGCCCTCCATGTGTGTATACAGGTGGCAGATGGGAGGGCAGGTAAGATAAAGTGTCTGTTGTTGACAAAAG
GGATCTCAGGCTCTCCAGCACCCATACCCTGCATCTACCCACAAGCAGAACAGCCACATACTGGTCCAGCCAGAAAAAG
CTGATTCAGCTCCAGTTTCTCTGGGATTATAACTTTATCTTCGACCATACTCTTCAGAAGTTGAGGTGGGGCCACGGCCA
AGGCTTTCTTCCACTTGGAAAGAAGTTCCCTCCCTTGATCGTCTCCAAACCCTTTGAAAGTTTACTGGAACCCAAATGAG
GCCTGGGGGTAAGGAGAGGGGGCCTCCAAGGACTCCTAGTCCAGCGCTCCTTCTGGTCCCACGCAATCTaTCCAAGTGG
TGCACACTGAGGGTTGGGACTGAGACCTAAAAAATAATAGAGTGTTCATCTGCTTGATCTGTTTGGTTTGCATTTAAGAAAC**ACAA**

Disease Models & Mechanisms • Supplementary information

TGGAGTACAGACAGACCGTTGGAGATGGGACTCTATTGTTCCTATGTCCCCT

5. PCR detection on homologous arms integration into GDNA in GNRH1, (Figure 1 B)

Touchdown PCR of GNRH1 homology arms

5p: 5ha-GNRH1-BamHI-F 5ha-GNRH1-NheI-Rv

3p: 3ha-GNRH1-XbaI-R 3ha-GNRH1-AscI-F

	1x (uL)	13x	Thermal cycle	5p	3p	
5x HF buffer	4,0	52,0	1x	98ºC	98ºC	3'
dNTPS(2.5mM)	1,6	20,8	8x	98ºC	98ºC	10''
DMSO(100%)	0,2	2,6	down 0.5ºC/cycle	73-69ºC	67-63ºC	30''
Fw(10uM)	1,0	13,0		72ºC	72ºC	20''
Rv(10uM)	1,0	13,0	30x	98ºC	98ºC	10''
Phusion	0,2	2,6		69ºC	63ºC	30''
Betaine	4,0	52,0		72ºC	72ºC	20''
H20	7,0	91,0	1x	72ºC	72ºC	8'
DNA	1,0					
TOTAL	20,0	á 19				

Disease Models & Mechanisms • Supplementary information

Supplementary references

References described in Table S3.

1. Lintas C, Persico AM. Neocortical RELN promoter methylation increases significantly after puberty. Neuroreport. 2010 Jan 27;21(2):114-8.

2. Simavli S, Thompson IR, Maguire CA, Gill JC, Carroll RS, Wolfe A, et al. Substance p regulates puberty onset and fertility in the female mouse. Endocrinology. 2015 Jun;156(6):2313-22.

3. Maguire CA, Song YB, Wu M, Leon S, Carroll RS, Alreja M, et al. Tac1 Signaling Is Required for Sexual Maturation and Responsiveness of GnRH Neurons to Kisspeptin in the Male Mouse. Endocrinology. 2017 Jul 1;158(7):2319-29.

4. Vastagh C, Rodolosse A, Solymosi N, Liposits Z. Altered Expression of Genes Encoding Neurotransmitter Receptors in GnRH Neurons of Proestrous Mice. Front Cell Neurosci. 2016 Oct 7;10:230.

5. Day FR, Thompson DJ, Helgason H, Chasman DI, Finucane H, Sulem P, et al. Genomic analyses identify hundreds of variants associated with age at menarche and support a role for puberty timing in cancer risk. Nat Genet. 2017 Jun;49(6):834-41.

6. Wray S, Grant P, Gainer H. Evidence that cells expressing luteinizing hormone-releasing hormone mRNA in the mouse are derived from progenitor cells in the olfactory placode. Proc Natl Acad Sci U S A. 1989 Oct;86(20):8132-6.

7. Chung WC,J., Moyle S,S., Tsai,Pei-San. Fibroblast Growth Factor 8 Signaling through Fibroblast Growth Factor Receptor 1 Is Required for the Emergence of Gonadotropin-Releasing Hormone Neurons. Endocrinology. 2008;149(10):4997-5003.

8. Kramer PR, Guerrero G, Krishnamurthy R, Mitchell PJ, Wray S. Ectopic expression of luteinizing hormone-releasing hormone and peripherin in the respiratory epithelium of mice lacking transcription factor AP-2alpha. Mech Dev. 2000 Jun;94(1-2):79-94.

9. Witchel SF, White C, Libman I. Association of the -243 A-->G polymorphism of the glutamate decarboxylase 2 gene with obesity in girls with premature pubarche. Fertil Steril. 2009 May;91(5):1869-76.

10. Kokay IC, Petersen SL, Grattan DR. Identification of prolactin-sensitive GABA and kisspeptin neurons in regions of the rat hypothalamus involved in the control of fertility. Endocrinology. 2011 Feb;152(2):526-35.

11. Nishimura I, Ui-Tei K, Saigo K, Ishii H, Sakuma Y, Kato M. 17beta-estradiol at physiological concentrations augments Ca(2+) -activated K+ currents via estrogen receptor beta in the gonadotropin-releasing hormone neuronal cell line GT1-7. Endocrinology. 2008 Feb;149(2):774-82.

12. Burger LL, Vanacker C, Phumsatitpong C, Wagenmaker ER, Wang L, Olson DP, et al. Identification of Genes Enriched in GnRH Neurons by Translating Ribosome Affinity Purification and RNAseq in Mice. Endocrinology. 2018 Apr 1;159(4):1922-40.

13. Aguillon R, Batut J, Subramanian A, Madelaine R, Dufourcq P, Schilling TF, et al. Cell-type heterogeneity in the early zebrafish olfactory epithelium is generated from progenitors within preplacodal ectoderm. Elife. 2018 Jan 2;7:10.7554/eLife.32041.

14. Pinilla L, Pineda R, Gaytan F, Romero M, Garcia-Galiano D, Sanchez-Garrido MA, et al. Characterization of the reproductive effects of the anorexigenic VGF-derived peptide TLQP-21: in vivo and in vitro studies in male rats. Am J Physiol Endocrinol Metab. 2011 May;300(5):E837-47.

15. Yang C, Ye J, Liu Y, Ding J, Liu H, Gao X, et al. Methylation pattern variation between goats and rats during the onset of puberty. Reprod Domest Anim. 2018 Jun;53(3):793-800.

16. Quaynor SD, Bosley ME, Duckworth CG, Porter KR, Kim SH, Kim HG, et al. Targeted next generation sequencing approach identifies eighteen new candidate genes in normosmic hypogonadotropic hypogonadism and Kallmann syndrome. Mol Cell Endocrinol. 2016 Dec 5;437:86-96.

17. Kim JH, Seo GH, Kim GH, Huh J, Hwang IT, Jang JH, et al. Targeted Gene Panel Sequencing for Molecular Diagnosis of Kallmann Syndrome and Normosmic Idiopathic Hypogonadotropic Hypogonadism. Exp Clin Endocrinol Diabetes. 2018 Sep 14.

18. Passemard S, Titomanlio L, Elmaleh M, Afenjar A, Alessandri JL, Andria G, et al. Expanding the clinical and neuroradiologic phenotype of primary microcephaly due to ASPM mutations. Neurology. 2009 Sep 22;73(12):962-9.

19. Salian-Mehta S, Xu M, Knox AJ, Plummer L, Slavov D, Taylor M, et al. Functional consequences of AXL sequence variants in hypogonadotropic hypogonadism. J Clin Endocrinol Metab. 2014 Apr;99(4):1452-60.

20. Salian-Mehta S, Xu M, Wierman ME. AXL and MET crosstalk to promote gonadotropin releasing hormone (GnRH) neuronal cell migration and survival. Mol Cell Endocrinol. 2013 Jul 15;374(1-2):92-100.

21. Zhu H, Shah S, Shyh-Chang N, Shinoda G, Einhorn WS, Viswanathan SR, et al. Lin28a transgenic mice manifest size and puberty phenotypes identified in human genetic association studies. Nat Genet. 2010 Jul;42(7):626-30.

22. Jayakody SA, Andoniadou CL, Gaston-Massuet C, Signore M, Cariboni A, Bouloux PM, et al. SOX2 regulates the hypothalamic-pituitary axis at multiple levels. J Clin Invest. 2012 Oct;122(10):3635-46.

23. Provenzano C, Pascucci B, Lupari E, Civitareale D. Large scale analysis of transcription factor TTF-1/NKX2.1 target genes in GnRH secreting cell line GT1-7. Mol Cell Endocrinol. 2010 Jul 29;323(2):215-23.

Disease Models & Mechanisms • Supplementary information
